# Quantitative and Computational Spinal Imaging in Neurodegenerative Conditions and Acquired Spinal Disorders: Academic Advances and Clinical Prospects

**DOI:** 10.3390/biology13110909

**Published:** 2024-11-07

**Authors:** Mary Clare McKenna, Jana Kleinerova, Alan Power, Angela Garcia-Gallardo, Ee Ling Tan, Peter Bede

**Affiliations:** 1 Computational Neuroimaging Group, Trinity College Dublin, 152-160 Pearse St, 2 D02 R590 Dublin, Ireland; 2 Department of Neurology, St James’s Hospital, James St, 8 D08 NHY1 Dublin, Ireland

**Keywords:** spinal cord, MRI, biomarker, radiology, neurodegenerative conditions, ALS, Parkinson’s disease, ataxia, diffusion tensor imaging, hereditary spastic paraparesis

## Abstract

**Introduction:** Quantitative spinal cord imaging has facilitated the objective appraisal of spinal cord pathology in a range of neurological conditions both in the academic and clinical setting. Diverse methodological approaches have been implemented, encompassing a range of morphometric, diffusivity, susceptibility, magnetization transfer, and spectroscopy techniques. Advances have been fueled both by new MRI platforms and acquisition protocols as well as novel analysis pipelines. The quantitative evaluation of specific spinal tracts and grey matter indices has the potential to be used in diagnostic and monitoring applications. The comprehensive characterization of spinal disease burden in pre-symptomatic cohorts, in carriers of specific genetic mutations, and in conditions primarily associated with cerebral disease, has contributed important academic insights. **Methods:** A narrative review was conducted to examine the clinical and academic role of quantitative spinal cord imaging in a range of neurodegenerative and acquired spinal cord disorders, including hereditary spastic paraparesis, hereditary ataxias, motor neuron diseases, Huntington’s disease, and post-infectious or vascular disorders. Results: The clinical utility of specific methods, sample size considerations, academic role of spinal imaging, key radiological findings, and relevant clinical correlates are presented in each disease group. **Conclusions:** Quantitative spinal cord imaging studies have demonstrated the feasibility to reliably appraise structural, microstructural, diffusivity, and metabolic spinal cord alterations. Despite the notable academic advances, novel acquisition protocols and analysis pipelines are yet to be implemented in the clinical setting.

## Simple Summary

This review examines the clinical and academic role of quantitative spinal cord imaging in neurodegenerative and acquired spinal cord disorders. Cervical spinal cord atrophy is readily detected in a range of motor neuron disease (MND) phenotypes (ALS, PLS, PMA, SBMA, PPS, and SMA), hereditary ataxias (SCA, FDRA), HSP, and other neurodegenerative disorders. Cord changes may be detected in the pre-symptomatic stages of the disease in association with certain genotypes. Longitudinal studies often capture progressive cord atrophy over time. Diffusion tensor imaging studies have detected cervical spinal cord diffusivity alterations in ALS, PLS, SBMA, FDRA, HSP, ALD, HAM/TSP, following spinal cord infarcts or sensory neuronopathies. The few MRS studies reveal altered metabolite ratios in the cervical cord in ALS and FDRA. These academic observations have important clinical implications, helping to define the potential diagnostic and monitoring role of these metrics and ultimately the development of clinically useful biomarkers for clinical trials, diagnostic applications, and tools to monitor disease progression.

## 1. Introduction

Recent methodological advances in quantitative spinal cord imaging have facilitated the objective appraisal of spinal cord pathology across a spectrum of genetic and acquired neurological conditions. Given the vast array of imaging techniques, for discussion purposes, spinal imaging methods may be categorized into structural, microstructural, or metabolic. Structural imaging methods include spinal cord cross-sectional area (CSA) measurements, which is a surrogate marker for whole spinal cord atrophy. It is estimated over a representative number of T1- (T1w) or T2-weighted (T2w) axial images at specific vertebral levels. Recent spinal cord segmentation methods have permitted selective appraisal of cervical cord grey matter (GM) and white matter (WM). Other T1w- or T2w-derived structural metrics include spinal cord eccentricity measurements. Microstructural imaging methods encompass diffusion tensor imaging (DTI), magnetization transfer (MT), and inhomogeneous magnetization transfer (ihMT) imaging. DTI-derived metrics—fractional anisotropy (FA), radial diffusivity (RD), axial diffusivity (AxD), and mean diffusivity (MD)—have been associated with different aspects of white matter integrity [1,2]. Novel MT and ihMT-derived metrics reflect primarily on myelination. The spinal implementation of novel white matter techniques such as neurite orientation dispersion and density imaging (NODDI), high angular resolution diffusion imaging (HARDI), and q-ball imaging (QBI) is ongoing [3,4,5,6]. Metabolic imaging methods include magnetic resonance spectroscopy, which measures voxel-wise neurometabolite concentrations or their ratios. The most commonly evaluated metabolites include N-acetyl aspartate (NAA) which is regarded as a marker of neuronal integrity; creatine (Cr), a proxy of tissue energy metabolism; choline (Cho), a membrane integrity marker; and myo-inositol (m-Ins), which is primarily associated with glial function. With the exception of special indications, spinal PET is seldom utilized [7], and spinal FDG-PET is typically reserved for distinguishing inflammatory myelopathies from neoplastic myelopathies [8]. Spinal functional MRI (fMRI) is also in its infancy compared to cerebral fMRI, but spinal fMRI has already proven to be a reliable measure of spinal activation in both resting state and task-based protocols [9]. Structural, microstructural, and metabolic spinal imaging methods generate complimentary information that helps to characterize the topography, extent, and nature of spinal cord involvement. While these spinal imaging methods are increasingly used in the academic setting and have enhanced our understanding of neurodegenerative processes in a range of neurological conditions, they are seldom utilized in the clinical setting. Accordingly, the main objective of this paper is to review advances in quantitative spinal imaging, identify barriers to their clinical implementation, and evaluate their potential role in clinical decision making, diagnosis, and monitoring. Additional aims include the discussion of stereotyped study limitations, identification of gaps in the literature, and evaluation of the biomarker potential of specific imaging techniques in clinical and clinical trial settings. As this is a dynamically evolving and ever-changing field of research, new spinal studies are published every day. Ultimately, we wish to raise awareness of emerging methods while candidly discussing their advantages and drawbacks.

## 2. Methods

**Figure 1:**
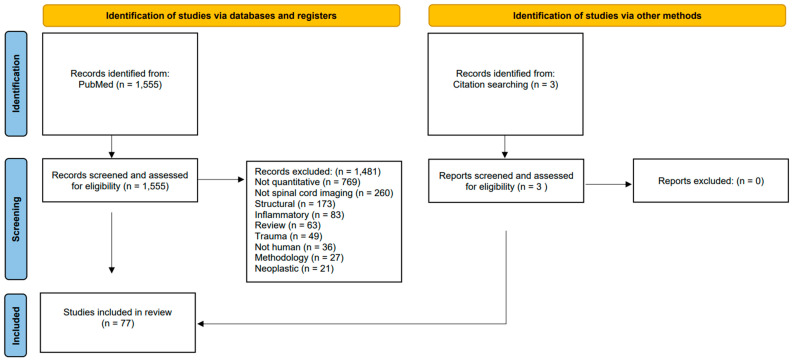
A PRISMA (Preferred Reporting Items for Systematic Reviews and Meta-analyses) flowchart outlining the study identification, screening, inclusion, and exclusion review process.

A scoping review was conducted using the PubMed repository (last accessed on 6 April 2023) in accordance with the “preferred reporting items for systematic reviews and meta-analyses” (PRISMA) guidelines (Supplementary File S1 and Figure 1). As this is not a systematic review, the review was not formally registered, and no dedicated protocol was generated. The following search strategy was used: (“Spinal Cord” OR “Cervical Cord”) AND (“Magnetic resonance imaging” OR “MRI” OR “DTI” OR “diffusion tensor imaging” OR “MRS” OR “magnetic resonance spectroscopy”) AND (“Neurodegenerative” OR “Neuromuscular” OR “Motor neuron disease” OR “primary lateral sclerosis” OR “PLS” OR “ALS” OR “amyotrophic lateral sclerosis” OR “MND” OR “SBMA” OR “spinobulbar muscular atrophy” OR “Kennedy’s disease” OR “spinal muscular atrophy” OR “SMA” OR “hereditary spastic paraparesis” OR “hereditary spastic paraplegia” OR “HSP” OR “Parkinson’s disease” OR “Parkinson disease” OR “Huntington’s disease” OR “Huntington disease” OR “Spinocerebellar ataxia” OR “SCA” OR “Friedreich’s Ataxia” OR “Friedreich ataxia” OR “Subacute combined degeneration” OR “Spinal cord ischemia” OR “Spinal cord infarct*” OR “tropical spastic paraparesis” OR “poliomyelitis” OR “HIV myelitis” OR “HIV myelopathy” OR “HIV vacuolar myelopathy” OR “ganglionopathy” OR “sensory neuronopathy”). The database search was limited to studies written in English and only involving human participants. A single reviewer (MCMcK) individually screened the 1555 abstracts for eligibility. All original research articles that investigated quantitative spinal cord imaging in neurodegenerative, neuromuscular, vascular, or infectious disorders were included. Opinion pieces, meta-analyses, and methodology papers were excluded. Previous review papers were also excluded, but the references of review papers of specific neurological conditions were screened for original research papers [10,11]. Structural, inflammatory, neoplastic, and traumatic spinal cord disorders were also excluded. Identified original research articles were systematically reviewed for the primary diagnosis, sample size, availability of genetic information, study design (cross-sectional/longitudinal), imaging methods, and the main quantitative spinal cord imaging results. A total of 77 studies were included (Figure 1). The results of these studies are next discussed stratified by clinical diagnosis.

## 3. Results

### 3.1. Motor Neuron Disease

#### 3.1.1. Amyotrophic Lateral Sclerosis

Amyotrophic lateral sclerosis (ALS) is a progressive neurodegenerative disorder primarily associated with motor cortex, brainstem, and anterior horn of the spinal cord degeneration [12,13,14,15,16,17], but frontotemporal, cerebellar, and subcortical involvement is increasingly recognized [18,19,20,21,22]. It is the most common type of motor neuron disease (MND). Clinical phenotypes of ALS can be defined by a multitude of criteria [23,24,25,26,27,28], but typically distinguished either (1) based on disease onset, such as limb onset, bulbar onset, or respiratory onset, (2) based on family history, such as sporadic or familial, (3) based on the degree of comorbid cognitive and behavioral impairment [23,29,30,31], or (4) by the predominance of upper versus lower motor neuron symptoms [32]. Sexual dimorphism in ALS is well recognized based on clinical, genetic, and imaging observations [33,34] and cerebral imaging studies routinely control for sex; the impact of sex on cord metrics is less well established. A multitude of spinal imaging cues have been associated with ALS, such as the “owl’s eyes” (snake eyes) phenomena at the anterior horns or high signal in the lateral columns along the corticospinal tracts, but these qualitative cues are not specific to ALS and may be observed in a range of neurological conditions [35]. The most common quantitative spinal imaging modalities in ALS include cervical cord area or volume estimation (66%; 21/32) [36,37,38,39,40,41,42,43,44,45,46,47,48,49,50,51,52,53,54,55,56], followed by diffusivity (47%; 15/32) [46,47,48,49,50,51,52,53,54,57,58,59,60,61,62], magnetization transfer ratio (MTR) (19%; 6/32) [48,49,50,51,52,55], T2 hyperintensities (13%; 4/32) [53,54,63,64]; spectroscopy (9%; 3/32) [65,66,67], and a single study used inhomogeneous magnetization transfer ratio (ihMTR) assessments (3%; 1/32) [52]. Several of these studies are multimodal (31%; 10/32) [46,47,48,49,50,51,52,53,54,55]. Most studies were performed on three Tesla MRI platforms (75%; 24/32). All studies appraised the cervical spinal cord, and a single study evaluated the entire spinal cord [43]. The mean number of participants was 33 (1–158), and the mean disease duration was 27 (7–77) months. Some studies availed of complementary genetic data (38%; 12/32) that ranged from case-by-case testing to systematically testing all cases for common familial ALS genetic mutations. The majority of spinal studies in ALS are cross-sections, but insightful longitudinal studies have been published (25%; 8/32) [39,40,45,46,47,50,53,56] with a mean follow-up duration of 9 months (3–18). A few particularly elegant pre-symptomatic spinal studies have been published [36,47,66] describing cord involvement before symptom manifestation in certain genotypes. No studies had accompanying post-mortem data to validate and explore the histopathological correlates of their in vivo radiological findings. The characteristics of existing quantitative spinal studies in ALS are summarized in Table 1. The consensus finding of spinal studies in ALS is the presence of progressive [39,40,45,50,53,56] cervical cord atrophy [37,38,39,40,41,43,44,45,46,49,50,51,53,54,56] compared to controls. Cord atrophy is thought to be driven by corticospinal tract (CST) degeneration in the cervical cord [43,44]. This is thought to be supported by an ultra-high-field 7-Tesla MRI study of the cervical cord in ALS [64]. Spinal cord GM and WM segmentation has consistently identified concomitant grey- and white-matter degeneration in the cervical cord [37,38,41,52]. This has recently been facilitated by novel acquisition protocols such as the phase-sensitive inversion recovery (PSIR) MR sequence that minimizes motion sensitivity and susceptibility [37,41]. The clinical correlates of spinal degeneration have been explored by several studies. A cross-sectional study that stratified patients according to King’s staging system reported GM atrophy in King’s stage 1, followed by progressive GM and WM atrophy in all cervical cord segments, increasing in a caudal direction in subsequent King’s stages [41]. This study predicted that the earliest detectable changes occur in the GM at the C3-C4 level and may even be detected several months before symptom onset [41]. A study evaluating the entire spinal cord in ALS identified the most marked radiological changes at C4-C7, and no significant atrophy was detected at thoracolumbar levels, suggesting that future ALS studies should focus on the cervical cord [43]. Longitudinal studies suggest that cervical cord atrophy is progressive in ALS [45], and grey matter volume reductions readily differentiate ALS from controls [38]. Reports on anterior-posterior diameter [63] and mean cross-sectional area (CSA) [42] alterations are less consistent in ALS, probably due to sample size limitations [42] and the low spatial resolution of input imaging data [63]. No significant differences are typically reported between disease-onset phenotypes [41], but a trend for more marked cervical cord involvement was noted in those with upper-limb onset [40,46] or upper-limb involvement [41,43] compared to other phenotypes. Bulbar-onset ALS seemed to be the least affected [37,46]. With regards to ALS genotypes, cervical cord atrophy has been demonstrated in *SOD1* [55], *VAPB* [36], and *C9orf72* [39,47] hexanucleotide repeat expansion carriers. Cord changes have been detected in pre-symptomatic mutation carriers long before projected symptom onset [36,47]. Overall cervical cord atrophy was detected in pre-symptomatic *VAPB* carriers [36], and cervical cord WM was atrophy identified in pre-symptomatic *C9orf72* hexanucleotide repeat expansion carriers [47]. The later cohort did not exhibit progressive GM or WM cord changes at 18-month follow-up [47], and similarly, no longitudinal changes were detected in another cohort of *C9orf72* carriers at short interval follow-up [39]. Fractional anisotropy reductions are the most common diffusivity finding in cervical cord DTI studies in ALS [46,51,54,58,59,61], often with a caudal predominance [49,59,62]. Central cord [62], anterior [46,58,62], lateral [46,49,52,57,58,60,62] and posterior [46,49,62] column diffusivity changes have been captured. This may be accompanied by increased RD [46,49,59,60], increased MD [46,60], or reduced AD [52] in the lateral CSTs and the posterior columns. However, significant MD [59,61] or AxD [46,59] changes are not always detected. The findings of longitudinal spinal cord DTI studies in ALS differ considerably depending on their follow-up interval: no significant changes are detected at 6 months [46]; a decline in FA at 9 months [53]; increased MD at 9 months [53] and 1 year [46]; and increased RD at 1-year follow-up [46]. With regards to ALS phenotypes, there was a trend to more marked FA reductions in upper-limb onset ALS [46], with bulbar-onset ALS being the least affected [46,61]. Regarding ALS genotypes, reduced FA is captured in pre-symptomatic *C9orf72* [47] and symptomatic *SOD1* [49] carriers. The pre-symptomatic phase of *C9orf72* has considerable clinical relevance [68,69,70] as it may represent an important window for early pharmacological intervention [71,72], and indeed, emerging pre-symptomatic spinal has shown progressive FA decline along the corticospinal tracts (CST) [47]. In a subset of pre-symptomatic *C9orf72* mutation carriers aged over 40 with a family history of ALS, reduced FA in the CSTs was also detected at baseline [47], suggesting that CST FA alterations in *C9orf72* carriers may help to identify those who are more likely to convert to ALS rather than frontotemporal dementia [47]. Spectroscopically, reduced NAA/Cr [65,66,67], NAA/m-Ins [65,66,67], NAA/Cho [66], and Cho/Cr [67] ratios and increased m-Ins/Cr [65] ratios are typically detected in the cervical cord in ALS. A simplistic but potentially helpful interpretation of MRS findings is that reduced NAA indicates neuronal loss, increased Cho levels suggest inflammation [65], and increased m-Ins represents gliosis [65]. A strikingly similar cervical cord metabolic pattern was also captured in pre-symptomatic *SOD1* carriers, i.e., reduced NAA/Cr, NAA/m-Ins, and m-Ins/Cr ratios [66], suggesting that early radiological metabolic changes may precede clinical or neurophysiological changes [66]. Cervical cord MTR is much less frequently evaluated in ALS, but progressively [50] reduced MTR ratios [48,49,50,51,52] are consistently observed, particularly in the lateral corticospinal tracts [49,50,52]. In a study of *SOD1* ALS, no significant MTR ratio alterations were detected [55]. A study investigating cervical cord ihMTR in ALS detected significantly reduced ihMTR in the WM, anterior GM, CSTs, and posterior columns [52]. The authors of this study suggested that ihMTR may be more sensitive at detecting microstructural changes than conventional MTR or DTI metrics, but this needs to be validated in multimodal studies [52]. Spinal imaging methods may be combined to differentiate patients with ALS from controls [46,48]. A multi-modal classification model using cervical cord CSA, DTI, and MTR variables accurately differentiated ALS from controls with a sensitivity of 88% and a specificity of 85% (AUC 0.96). The best-performing individual variables were RD, followed by FA, and then CSA at C5 spinal level [48]. Multi-modal cervical cord imaging data may also be used to develop prognostic models [38,51]. The risks of direct clinico-radiological associations are well described [73], but structural cervical cord measures have been correlated to muscle strength [49,50], respiratory function [56], disease duration [41,44,55], and motor disability [38,40,44,45,46,50,52,55]. The decline in motor function is thought to be associated with cervical cord atrophy in sporadic ALS but not in those with *C9orf72* [39]. The level of predominant cervical cord atrophy anatomically corresponds with muscle weakness patterns [49]. WM and GM cervical cord measures both correlate independently with disease severity [41,46]. Cord diffusivity metrics in ALS also correlate with motor function [59], muscle strength [52], respiratory involvement [59], disease duration [52], and disease severity [46,49,50,54,59]. FA reductions in particular, either of the entire cervical cord [46,52,54,59] or lateral columns [49,50], correlate with muscle strength [52,59], disease severity [46,49,50,54], and rate of disease progression [61]. Reduced NAA/Cr and NAA/m-Ins ratios evaluated by MRS are associated with respiratory involvement and disease severity [65,67]. MTR and ihMTR metrics also correlate with muscle strength and disease duration [52]. Notwithstanding these associations, structural [36,37,39,53,54], diffusivity [53,57,58,60], or metabolic [66,67] metrics often show no correlations with clinical variables [73]. In summary, the main quantitative spinal cord imaging metrics used in ALS-related studies are cross-sectional area measurements and diffusivity metrics. Alterations in these metrics can be captured in pre-symptomatic mutation carriers and correlate well with clinical measures in the symptomatic phase of the disease. Longitudinal spinal studies in ALS readily capture dynamic pathological changes over time [45,50,56], including progressive MTR, structural, and diffusivity alterations [45,50,53]. While changes in these metrics align with our current understanding of ALS pathophysiology, they are not incorporated as auxiliary outcome measures in current clinical trial designs. The practical limitation of using these metrics in ALS studies lies in the difficulty of acquiring MRI data in patients with respiratory weakness and bulbar symptoms, i.e., scanning patients with ALS is increasingly challenging as the disease progresses.

#### 3.1.2. Primary Lateral Sclerosis

Primary lateral sclerosis (PLS) is a low-incidence MND subtype characterized by exclusive upper motor neuron degeneration [74,75,76]. It typically presents as a gradual onset of lower limb stiffness and spasticity [75,77]. Imaging studies of PLS typically focus on motor system degeneration [78,79,80,81], but subcortical, cerebellar, and frontotemporal changes have been described more recently [18,82,83,84,85]. Despite the marked lower limb spasticity associated with PLS, spinal cord studies are sparse, and with the exception of a few cross-sectional [86] and longitudinal studies [39,40], not many spinal PLS studies have been published. These studies evaluated spinal cord area [39,40], diffusivity [86], and implemented myelin water imaging (MWI) using gradient and spin echo sequence (GRASE) [86]. Published PLS spinal studies are typically rather small with 2–18 participants, and patients are sometimes merged with larger ALS cohorts [40] (Table 1). In an admixed MND cohort incorporating mostly ALS and a few PLS patients, there was cervical spinal cord atrophy with a trend towards longitudinal progression at 6-months follow-up [40]. The presence of cervical cord atrophy was confirmed in a small cohort of PLS patients, but no longitudinal changes were noted at follow-up 6 months later [39]. A DTI study identified increased RD in the cervical cord GM and FA reductions in the lateral columns [86], and GRASE MWI detected low myelin water fraction in the lateral columns along the CSTs [86]. Baseline cervical cord CSA correlated with disability as measured by ALSFRS-R in a cohort of PLS patients [39] and in a clinically admixed MND group consisting of both of ALS and PLS [40]. Disease progression measured by functional rating scale-score decline also correlates with cervical cord atrophy in PLS [39].

#### 3.1.3. Progressive Muscular Atrophy

Progressive muscular atrophy (PMA) is an MND phenotype characterized by exclusive lower motor neuron degeneration. It presents clinically with progressive muscle wasting and weakness and is associated with a better prognosis than ALS. There are two quantitative spinal cord imaging studies that specifically evaluate this condition (Table 1). One study refers to this cohort as “sporadic adult onset lower MND” [63]. The initial 1.5 T MRI cross-sectional study did not detect any changes in cervical spinal cord morphology or signal alterations in PMA compared with controls [63]. In contrast, a recent 3.0 T MRI longitudinal study detected progressive upper cervical cord atrophy in PMA over a median follow-up of 5.5 (3–59) months [39]. Cervical cord atrophy in PMA correlated with functional decline (change in ALSFRS-R) but not with disease severity (ALSFRS-R score) [39].

#### 3.1.4. Spinal and Bulbar Muscular Atrophy

Spinal and bulbar muscular atrophy (SBMA), also known as Kennedy’s disease, is an X-linked autosomal recessive MND caused by a trinucleotide repeat in the *AR* gene [87,88,89]. It typically presents with insidious-onset bulbar dysarthria and dysphagia with weakness and wasting of the proximal extremities [87,88]. It is often associated with features of androgen insensitivity, such as gynecomastia and a range of endocrine, metabolic, and cardiac manifestations [87]. The radiological involvement of the spinal cord in SBMA has been explored in two cross-sectional studies that primarily investigated ALS but also included patients with SBMA (Table 1). The mean number of genetically confirmed participants was 12.5 (6–19) with a mean symptom duration of 20.25 (16.5–24) years. In SBMA, there is cervical and thoracic spinal cord atrophy compared to both healthy controls and patients with ALS [63]. This may have been because of statistically longer disease duration in the SBMA cohort when compared to the ALS cohort [63]. Spinal cord atrophy in SBMA is postulated to be driven by marked dorsal column involvement [63]. Interestingly, cervical cord diffusivity metrics do not differ significantly between patients with SBMA and controls [61].

#### 3.1.5. Post-Polio Syndrome

Post-polio syndrome (PPS) is a condition that may develop several decades after the initial polio infection [90,91]. It often presents as generalized fatigue, progressive muscle weakness, and atrophy, yet recent imaging studies captured no evidence of significant cerebral atrophy [92,93,94]. A cross-sectional case-control imaging study investigated spinal cord involvement in PPS [95] (Table 1) and detected reduced cervical and thoracic spinal cord area in PPS compared to controls. Cord atrophy was more marked in those with progressive disease, and cord metrics correlated with muscle strength in the corresponding myotomes. These findings were interpreted by the authors as evidence of a secondary post-infectious secondary neurodegenerative process [95].

#### 3.1.6. Spinal Muscular Atrophy

Spinal muscular atrophy (SMA) is an autosomal recessive neuromuscular disorder that is caused by mutations in the *SMN1* gene. It presents with gradually progressive muscle weakness involving the arms, legs, and respiratory muscles. The clinical phenotype is stratified according to disease severity (in decreasing order from type 0-type IV) and age of onset [96,97]. There have been both cross-sectional [96,98,99] and longitudinal [97] imaging studies investigating cervical cord involvement in SMA (Table 1). All of these studies were conducted on 3.0 T MRI scanners and investigated spinal cord cross-sectional area [96,97,98,99]; and two studies also investigated DTI metrics in addition [96,99]. All participants had a genetically confirmed diagnosis, but the majority of studies focus on type III or IV clinical phenotype [96,97,98,99]; only a single participant had the more severe type II clinical phenotype [99]. The mean number of participants in the studies was 17 (10–25) with a mean symptom duration of 28 years. Cervical cord atrophy [96,98] with selective GM degeneration [96] is readily detected in SMA. Atrophy is most prominent in levels that innervate proximal muscles; C3-C6 [98]. The pattern of anterior-predominant cord atrophy is thought to indicate anterior horn cell degeneration [98]. Increased cervical cord GM AxD was noted [99], without other DTI abnormalities [96,99]. The only longitudinal SMA study captured no significant change in cervical cord GM or WM cross-sectional area over 2 years [97]. This may be due to the particularly slow disease process in the later stages of the disease or due to early degenerative changes without subsequent progression [97]. This observation could preclude the use of structural cervical cord as an objective biomarker in SMA clinical trials [97]. Similar to ALS imaging studies [73], clinico-radiological correlations have led to variable results. While one study identified significant associations between cervical cord metrics and deltoid muscle strength [96], others detected no correlations between clinical measures and imaging metrics [98,99].

## 4. Hereditary Ataxias

### 4.1. Autosomal Dominant Hereditary Ataxias

#### Spinocerebellar Ataxia

Spinocerebellar ataxias (SCA) encompass a heterogeneous group of neurodegenerative disorders with cerebellar symptom predominance but may be associated with other clinical features such as parkinsonism, pyramidal signs, peripheral neuropathy, or urinary dysfunction. Spinal cord involvement has been evaluated by both cross-sectional case-control studies and longitudinal studies spanning over 1–5 years [100,101] (Table 2). Existing spinal studies in SCAs exclusively evaluate the cervical cord, focusing primarily on spinal cord cross-sectional area [100,101,102,103,104,105,106,107] and eccentricity [102,103,104,106]. The majority of studies rely on 3.0 T MRI data [100,102,103,104,106], but data acquired on 1.5 T [105] and 0.5T [101] platforms have also been evaluated. The mean number of participants is 42 (7–210) in these studies, most of whom had a genetically confirmed diagnosis. None of these studies had accompanying post-mortem data available. The mean symptom duration in these studies was 9 years, and interestingly, two studies included pre-symptomatic cohorts [103,107]. Cervical cord atrophy and flattening was captured in SCA1 [101,104], SCA3 [101,102,103] and SCA7 [106]. It is hypothesized that this is primarily driven by posterior column [102,103] and spinocerebellar tract [107] degeneration. Cervical cord atrophy may be detected in the pre-symptomatic [103,107], early-symptomatic [105], and symptomatic phases of SCA3 [107]. A cross-sectional study of SCA3 detected a relatively linear progression in cervical cord atrophy in a cohort stratified for symptom duration (<5 years, 5–10 years, 10–15 years, and >15 years) [103]. These findings, however, were not validated by longitudinal studies [100,101]. It is thought that lack of progression over time is due to long-standing disease, and patients have already reached maximal spinal cord atrophy [100]. In SCA6, which is often considered a pure cerebellar degeneration phenotype, no cervical cord atrophy was detected [105]. Nonetheless, mean cervical cord CSA correlated with disease severity [105]. Cord indices in SCAs may [102,104,106] or may not [105] correlate with clinical parameters. The degree of cervical cord atrophy correlates with disability and disease duration in SCA1 [104], SCA3 [102], and SCA7 [106]. In some instances, clinico-radiological associations may outperform other imaging biomarkers—such as cerebellar or brainstem imaging metrics [104].

### 4.2. Autosomal Recessive Hereditary Ataxias

#### 4.2.1. Friedreich’s Ataxia

Friedreich’s ataxia (FDRA) is an autosomal recessive trinucleotide repeat expansion disorder that manifests clinically as progressive dysarthria, limb- and gait-ataxia, and loss of lower limb reflexes. It has extra-neurological manifestations, such as cardiac involvement. Radiologically, cerebral, cerebellar, and cervical cord atrophy are the hallmark findings. Spinal cord involvement in FDRA has been investigated by a number of cross-sectional [108,109,110,111,112] and longitudinal [113] imaging studies. These often primarily focus on cross-sectional area and eccentricity [108,109,110,111,112,113], but DTI studies [110,113] and an MRS study [113] have also been published (Table 2). All of these were conducted using 3.0 T platforms, and all participants had a genetically confirmed diagnosis of FRDA. Similar to spinal studies in other neurodegenerative conditions, no post-mortem data were reported to correlate radiological findings with post-mortem histology. The mean number of participants was 68 (21–256) with a mean disease duration of 12.5 years. Quantitative imaging studies have consistently demonstrated cervical and thoracic spinal cord atrophy and increased eccentricity in FDRA compared to healthy controls [108,109,110,111,112,113]. There is greater atrophy in the cervical cord; and greater anteroposterior flattening in the distal thoracic cord [109]. These findings may be captured in early disease [112]. It is suggested that this pattern indicates preferential degeneration of the dorsal columns, lateral CSTs, and spinocerebellar tracts [108,109,112]. DTI studies have captured FA reductions, increased RD, increased MD, and increased AxD [110,113] in the dorsal columns, fasciculus gracilis, fasciculus cuneatus, and corticospinal tracts [110]. An MRS study detected N-acetyl-aspartate (tNAA) reductions, increased m-Ins, and a decreased ratio tNAA/m-Ins in the cervical cord compared to healthy controls [113]. A longitudinal study demonstrated progressive spinal cord atrophy, tNAA/m-Ins ratio changes, and a decline in FA over time [113]. Longitudinal atrophy was confined to cord WM and not the GM [113]. There are promising initiatives to map these radiological changes systematically in FDRA in a multimodal setting, including spinal morphometric, diffusivity, and spectroscopy metrics [114]. Spinal indices show good correlation with clinical measures [108,109,110,112,113]. The cervical cord CSA correlates with disease duration [109,110] and disease severity as measured by Friedreich’s Ataxia Rating Scales (FARS) [108,110,112,113], Scale for Assessment and Rating of Ataxia (SARA) [109,113], Inventory of Non-Ataxia Signs [109], or Spinocerebellar Ataxia Functional Index (SCAFI) [109]. Tract-wise and total WM DTI [110,113] as well as MRS [113] correlate significantly with disease severity [110,113]. CST DTI metrics show correlations with disease duration [110]. Similar to ALS studies [115,116], the question of whether imaging findings represent developmental or neurodegenerative changes is often raised [108,109]. Recent studies suggest coexisting developmental alterations with active neurodegenerative processes [111,112,113]. Progressive cervical cord atrophy has been observed with stable preserved eccentricity [111,112] and the findings interpreted as degenerative CST and developmental dorsal column abnormalities [112]. A prospective longitudinal study has demonstrated progressive cervical spinal cord structural and metabolic changes [113]. While spinal cord CSA was proposed as a potential imaging biomarker to monitor disease progression, this needs to be confirmed on well-designed longitudinal studies [112].

#### 4.2.2. Autosomal Recessive Cerebellar Ataxia Type 1

Autosomal recessive cerebellar ataxia type 1 (ARCA1) is a progressive cerebellar syndrome caused by a mutation in the *SYNE1* gene. It may be associated with some degree of cognitive impairment and pyramidal signs. A cross-sectional case-control study did not detect cervical cord atrophy in a small cohort of ARCA1 compared with controls (Table 2). It was suggested that the presence or absence of cervical cord atrophy may be helpful to differentiate autosomal recessive ataxias, e.g., FDRA, but sample size limitations may preclude conclusive observations in that regard [117].

### 4.3. Hereditary Spastic Paraparesis

Hereditary spastic paraparesis (HSP) refers to a heterogenous group of neurodegenerative disorders. It may be classified according to the primary phenotype or the underlying genotype. “Pure-HSP” (pHSP) phenotype refers to a clinical presentation limited to progressive lower limb weakness and spasticity, and “complicated-HSP” (cHSP) phenotype involves other systems. There is a wealth of radiological evidence for spinal cord involvement in HSP (Table 3). The mean number of study participants in these studies is 18 (5–40), with a mean disease duration of 18 years. Most participants carried a genetically confirmed diagnosis [118,119,120,121,122,123,124,125,126,127,128]. For the purpose of comparative analyses, participants were either stratified by the clinical phenotype [118,127,129] or their genetic diagnosis [120,121,122,123,124,125,126]. Similar to other spinal studies in neurodegenerative conditions, no supporting post-mortem data are available. The majority of HSP studies evaluate spinal cord area and eccentricity [118,119,120,121,122,124,125,126,127,129], but diffusivity metrics have also been investigated [61,123,124,128]. All studies evaluated the cervical cord, with some also appraising the thoracic cord [119,121,122,123,124,127,129]. Most studies used a 3.0 T MRI scanner [120,121,122,123,125,126,128], some used a 1.5 T platform [118,119,124,127], and one study relied on data from a 1T scanner [129]. Cervical and thoracic cord atrophy is well recognized in both pHSP and cHSP [118,119,127]. In a cohort of pHSP, reduced anteroposterior thoracic cord diameter was noted in comparison to healthy controls [129]. Despite the distinctly different phenotypes, there were no detectable spinal differences between pHSP and cHSP [118,127]. No distinguishing DTI profiles were observed in a clinically heterogenous group of HSP patients as part of an ALS study [61]. In genetically defined cohorts, varying degrees of spinal cord atrophy are described in SPG4 [124,125,126], SPG5 [122,123], SPG6 [119], SPG8 [119], SPG11 [120,126]; sometimes in SPG3A [119,121,126]; but not in SPG7 [126]. Subtle spinal cord atrophy in SPG3A [119] may elude detection [126]. Cord atrophy is not typically associated with cord eccentricity changes [120,125] and is more pronounced in the thoracic cord [119,122]. DTI studies have captured tract-specific degeneration with reduced FA and increased RD in the pyramidal tracts and reduced FA in the dorsal columns in the cervical cord in a genetically heterogenous group of patients with HSP [128]. In SPG4, reduced FA was noted in the dorsal columns, lateral and ventral funiculi, and increased RD at the lower cervical and upper thoracic levels [124]. In SPG5, there was reduced FA, increased RD, and increased MD in the dorsal columns and lateral corticospinal tracts in the cervical and upper thoracic cord [123]. Spinal cord metrics in HSP may [120,124,126,128] or may not [118,119,123,126,127,128] correlate with clinical measures. Symptom duration and disability were linked to reduced cervical cord GM area in SPG4 [126] and reduced cervical cord CSA in SPG11 [120]. Disease severity was associated with FA in the lateral funiculi [124] and dorsal column RD [128] in SPG4. However, spinal cord atrophy does not always correlate with clinical metrics in clinically [118,127] or genetically defined HSP [119,126]. This may be due to the “ceiling effect” whereby participants are captured late in their disease process, with considerable disability and established spinal cord atrophy [118,126].

## 5. Other Genetic Neurodegenerative Disorders

### 5.1. Huntington’s Disease

Huntington’s disease (HD) is an autosomal dominant trinucleotide repeat expansion disorder manifesting in motor disability, psychiatric symptoms, and cognitive impairment. Cervical spinal cord involvement in HD has been evaluated by dedicated quantitative imaging studies (Table 4). These studies demonstrate progressive upper cervical cord cross-sectional area reductions in both early [130] and established [131] disease. Cervical cord atrophy may [131] or may not [130] be detected in pre-symptomatic cases. There are inconsistent reports of clinico-radiological associations between cervical cord measures and motor deficits in HD [130,131]. Similar to other neurodegenerative conditions [115,116,132], it is often questioned whether radiological changes represent developmental or degenerative changes. However, the association between progressive cord atrophy and worsening motor deficits would seem to indicate a neurodegenerative rather than developmental process [130,131].

### 5.2. Adrenoleukodystrophy

X-linked adrenoleukodystrophy (ALD) is a rare inborn error of metabolism that is caused by mutations in the *ABCD1* gene. It results in defective peroxisomal beta-oxidation, causing very long-chain fatty acid accumulation in plasma and tissues. It is sometimes referred to as “metabolic hereditary spastic paraplegia” or “adrenomyeloneuropathy” because it clinically presents as a spectrum of adult-onset adrenocortical insufficiency, progressive myelopathy, and peripheral neuropathy. Spinal cord imaging studies in ALD appraise both spinal cord area [133,134,135] and diffusivity metrics [133,134] (Table 4). The mean number of participants in these studies is 20 (6–42), with a mean symptom duration of 12.5 years. Longitudinal studies have a mean follow-up interval of 1.5 years [135]. One study also included pre-symptomatic participants [135]. Spinal cord studies in ALD have captured cervical and thoracic cord CSA reductions [133,134,135], which were more marked in the thoracic region [133,134]. Flattening of the cervical cord was interpreted as selective dorsal column degeneration [135]. Longitudinal studies capture a trend of progressive upper cervical [135] and upper thoracic [134] cord atrophy. There was no difference in cervical cord CSA in asymptomatic patients compared to controls [135]. DTI studies capture reduced FA [133,134], reduced AxD [133], and increased RD [133] in the upper cervical cord [133]. There is significantly reduced FA, increased MD, and increased RD in the upper cervical cord WM at 2-year follow-up [134]. Cervical cord atrophy may correlate with disease severity [135]; however, DTI metrics do not show associations with clinical measures [133].

## 6. Acquired Spinal Cord Disorders

### 6.1. Sensory Neuronopathy

Sensory neuronopathy is characterized by selective dorsal root ganglia degeneration manifesting in marked ataxia and sensory symptoms. It may be “idiopathic” or secondary to autoimmune, paraneoplastic, infectious, metabolic, toxic, or genetic causes. Standard clinical imaging may reveal non-enhancing T2 hyperintensities along the posterior columns on axial views. Only a few MRI studies have appraised these alterations quantitatively. A cross-sectional study evaluated DTI metrics [136]; and a longitudinal study assessed both cord morphology and signal intensity of the ganglia, posterior columns, and C7 nerve root [137] (Table 5). The mean number of participants was only 18 (9–28) [136,137], with a mean disease duration of 8 (4–11) years, encompassing diverse acquired etiologies. Decreased cross-sectional area and increased dorsal root ganglion and posterior column signal intensity and decreased C7 nerve root size were detected in sensory neuronopathy using multiple-echo data image combination (MEDIC) and coronal turbo inversion recovery magnitude (TIRM) sequences [137]. A single DTI study demonstrated cervical cord FA reductions at C3-C4 that differentiated patients with sensory neuronopathies from disease and healthy controls [136]. Interestingly, both the MEDIC posterior column hyperintensities [137] and reduced cervical cord FA [136] are observed in patients without the characteristic T2-weighted posterior column abnormalities on clinical imaging, even in those with short disease duration <1 year [136]. This demonstrates that novel MRI sequences may be more sensitive at detecting spinal cord involvement in sensory neuronopathy and confirming clinical diagnostic suspicions. Longitudinal observations in a single case suggest that radiological changes may begin in the nerve root and propagate towards the posterior columns [137]. The severity of radiological changes in sensory neuronopathies does not seem to correlate with disability [136,137]. Cervical cord FA reductions, however, correlated with pain scores (Leeds Assessment of Neuropathic Symptoms and Signs), indicating that sensory neuronopathy is indeed associated with neuropathic pain [136].

### 6.2. HTLV-1 Associated Myelopathy and Tropical Spastic Paraparesis

HTLV-1 associated myelopathy/tropical spastic paraparesis (HAM/TSP) is a post-infectious myelopathy that presents as a gradually progressive spastic paraparesis that may be associated with sphincter involvement and sensory disturbance. Typical spinal imaging features include spinal cord atrophy and increased signal in the lateral columns. This has been further evaluated in cross-sectional case-control quantitative imaging studies [138,139,140,141] (Table 5). Most studies used 3.0 T MRI platforms [139,140,141], and only a single study used 1.5 T MRI [138]. Spinal cord area [139,140,141], volumes [138,141], T2 hyperintensities [141], and diffusivity metrics [141] were evaluated in HAM/TSP. Most studies focused on the cervical and thoracic spinal cord [138,139,140,141], and only a single study investigated lumbar cord involvement [139]. One HAM/TSP study also included post-mortem data [140]. The mean number of symptomatic, “definite”, or “possible” HAM/TSP participants was 12.5 (7–18) with a mean symptom duration of 8.75 years, and the mean number of asymptomatic HTLV-1 carriers was 6 (2–11). In “definite” HAM/TSP, cervical [138,139,140,141], thoracic [138,139,140,141], and lumbar [139] spinal cord atrophy was identified compared to controls. This was demonstrated by both cross-sectional cord area [138,139,140,141] and volumetric measures [138,141]. The degree of volume loss was greater in the thoracic cord [138,141]. These observations were more pronounced in those with longer disease duration [141]. Spinal cord atrophy was confirmed pathologically; it was particularly prominent in the lateral columns [140]. In those with “possible” HAM/TSP, reduced thoracic cord volumes were noted very similar to those with “definite” HAM/TSP [138]. In asymptomatic HTLV-1 carriers, the spectrum of spinal cord atrophy ranged from normal [138,139,141] to intermediate between normal and “definite” HAM/TSP [139]. In “definite” HAM/TSP, focal T2 hyperintensities were demonstrated in the bilateral anterolateral and dorsal columns extending over several spinal segments [141]. DTI captured FA reductions in the ventral and dorsal spinal tracts compared to controls. No focal lesions or DTI abnormalities were detected in asymptomatic HTLV-1 carriers [141]. Spinal findings in HAM/TSP show associations with clinical metrics [139,141]; reduced cervical cord area [139,141] and volume [141] correlated with disease duration; reduced cervical and thoracic cord area correlated with the Ambulation Index (an ordinal scale based on the 25-foot timed walk test) [139]; and reduced FA in the dorsal tracts correlated with the American spinal cord injury association (ASIA) score [141]. Conversely, the imaging metrics did not correlate with clinical measures of disease severity in other studies [138,140].

### 6.3. Vascular Aetiologies

Spinal cord infarction is a relatively rare ischemic insult that may involve the anterior or posterior spinal cord arteries, manifesting in distinct clinical syndromes. Anterior spinal cord infarction presents with acute-onset back pain, bilateral lower limb weakness and numbness, sphincter disturbance, and relative sparing of proprioception and vibration. Posterior spinal cord infarction presents with unilateral sensory loss, including impaired proprioception and vibration. It may be “idiopathic” or secondary to atherosclerosis, trauma, or rare etiologies such as fibrocartilaginous embolism. It is radiologically characterized by abnormal T2 signals in the affected vascular territory, but these are often not detected in the acute setting. A longitudinal case series quantified dynamic FA variations in spinal cord infarction [142] (Table 5), revealing FA reductions in the acute setting [142], followed by decreasing FA in a case with worsening symptoms and increasing FA in a case with improving symptoms [142]. It was hypothesized that the younger age and possibly smaller infarct volume may have accounted for the clinical and radiological improvement in the latter case [142].

## 7. Discussion

Our review highlights that quantitative spinal cord imaging techniques not only offer important academic insights in a range of neurodegenerative and acquired spinal conditions but may soon be developed into viable biomarker applications with real-life clinical utility. Overall, the available literature suggests satisfactory detection sensitivity of clinically relevant pathology at relevant spinal levels in pathognomonic, disease-associated cord structures. The most striking observation is that there is a considerable gap between academic observations and clinical applications, i.e., the majority of the above quantitative cord imaging techniques are seldom utilized in the clinical setting despite their potential in informing clinical decisions, confirming suspected diagnoses, and monitoring disease progression objectively. We have therefore structured our discussion to review the main achievements of the field of quantitative spinal imaging, identify the shortcomings of recent studies, define the most urgent priorities for future research, and review the main barriers to implementing these techniques in routine clinical practice.

## 8. Academic Insights

Spinal cord imaging studies have enhanced our understanding of disease pathogenesis and propagation by characterizing anatomical patterns of spinal cord involvement from the pre-symptomatic to the terminal stages of neurodegenerative conditions. In some conditions, there is ongoing debate whether radiological changes represent developmental or neurodegenerative changes, or as argued by some neurodegenerative superimposed on developmental changes in some instances [111,112]. Our review has highlighted that computational spinal cord studies are primarily conducted in conditions like ALS, followed by HSP and then SCA, despite ample evidence of cord involvement in a range of other neurological conditions [35,87,90,143]. Overall, all of the reviewed papers confirm the feasibility of meaningfully assessing and measuring spinal changes in neurodegenerative and acquired spinal cord conditions. The main conceptual and academic achievements of the reviewed studies include the characterization of disease-associated disease burden patterns [46,49,52,57,58,60,62], (2) longitudinal trajectories including anatomical propagation patterns and rate of progression [46,53], (3) genotype-associated spinal signatures, and (4) pathological change in asymptomatic mutation carriers [47,69,70]. Several of the above studies also highlight that neurological conditions primarily associated with cerebral involvement, such as PD and HD, also exhibit detectable spinal cord pathology [130,144,145]. The demonstration of considerable cord changes in asymptomatic mutation carriers with no overt neurological disability [47] suggests a certain resilience to pathological change. The notion of network redundancy to withstand some degree of degenerative change without manifesting in motor symptoms is sometimes referred to as “motor reserve”. Analogous to the concept of “cognitive reserve” [146], the notion of “motor reserve” was initially coined based on cerebral studies and is an emerging field of research across a range of neurodegenerative conditions [147,148,149]. Many of the published academic studies have direct clinical relevance. Spinal FA alterations in pre-symptomatic *C9orf72* hexanucleotide repeat expansion carriers may help to predict phenoconversion to ALS rather than frontotemporal dementia [47]. Spinal spectroscopic changes may precede detectable structural alterations [66], highlighting its value as an early surrogate marker of neurodegenerative change. Cord ihMTR has been proposed to be more sensitive in detecting microstructural changes than conventional MTR or DTI metrics [52]. While this needs further validation, it highlights the value of multimodal academic studies where a panel of complementary biophysical metrics is evaluated so that their detection sensitivity and tracking potential of specific markers can be juxtaposed and the most relevant metrics are selected for clinical use.

## 9. Clinical Applications

Despite the considerable progress in demonstrating the potential of quantitative spinal imaging in neurodegeneration, there remains a considerable gap between academic and clinical imaging, and advances in spinal imaging have not been translated into routine clinical applications. Routine clinical spinal protocols continue to be optimized for quick acquisition times to meet the radiological demands of busy hospitals. Spinal imaging is often first acquired in the sagittal plane, and depending on local protocols, radiographers choose the relevant levels for axial views. While this may be satisfactory in structural etiologies such as disk protrusions or tumors, it is less ideal in neurodegenerative conditions. While the large slice gaps of clinical protocols reduce time of acquisition, important intensity changes or signal abnormalities may be missed. While in the academic setting cardiac and respiratory gating is routinely implemented, this is seldom utilized in standard clinical imaging. Notwithstanding these considerations, quantitative spinal imaging in the clinical setting has a huge potential, including (1) the confirmation of the suspected diagnosis, (2) distinguishing similar phenotypes such as ALS/PLS, (3) tracking accruing pathology over time, (4) assessing response to therapy, (5) serving as putative endpoints in clinical trials, (6) potentially acting as prognostic markers, and (7) conceivably, predicting phenoconversion in asymptomatic mutation carriers. There are promising academic studies heralding “real-life” clinical applications in the near future. The distinct radiological patterns of spinal cord involvement with tract-specific degeneration may be used as an adjunctive diagnostic tool. For example, structural imaging studies revealing spinal cord atrophy with increased eccentricity suggest preferential dorsal column degeneration, as seen in FDRA, SCA, and ALD, whereas spinal cord atrophy without increased eccentricity suggests preferential corticospinal tract degeneration, as seen in ALS and HSP. Cerebral MRI data from single individuals have been successfully interpreted in various classification models across a range of neurodegenerative conditions, including AD, MCI, PD, and ALS [150,151,152,153,154,155,156,157,158,159]. With a few exceptions [48], diagnostic classification models have not been widely used on spinal data sets. The lessons of brain-data-based individual-scan interpretation frameworks, which include various machine-learning (ML) and z-score-based approaches, should be carefully considered and applied to spinal data, or combined brain-spinal protocols should be developed to improve the categorization accuracy of existing models [160,161,162,163,164,165]. One of the key observations from cerebral studies is that high accuracy can be achieved in distinguishing a single disease group from healthy controls, but the distinction of two phenotypes based on MRI data alone is much more challenging. Similar to brain applications [166], quantitative spinal cord imaging data may also be ultimately used as an imaging biomarker in clinical trials. The currently used clinical scales are subject to inter-rater variability and may not capture changes in slowly progressive neurodegenerative disorders, whereas quantitative imaging metrics offer objective data that may precede these clinical changes. Ultimately, quantitative spinal data may be used to measure baseline disease burden, track disease progression, and evaluate response to therapy in clinical trials of disease-modifying therapies. This concept was demonstrated in a preliminary study of *SPG5* that identified T9 spinal cord area as a potential clinical trial primary endpoint; however, the proposed study duration of 14 years was too long to be applicable to real-world clinical trials [122]. The follow-up intervals of academic longitudinal imaging studies are often criticized for being too short to capture significant changes; however, short follow-up intervals reflect the real-world designs of clinical trials that seldom go on for several years, especially in rapidly progressive conditions such as ALS. Therefore, radiological markers that sensitively detect subtle change over short follow-up periods are of particular pragmatic utility. Despite the achievements of the above studies, the development of research protocols into viable clinical applications is still awaited.

## 10. Gaps and Shortcomings

Relatively stereotyped shortcomings can be identified in the current literature of quantitative spinal imaging. Spinal studies often suffer from limited sample sizes, and the small cohorts of imaging studies limit the reliability and generalizability of radiological findings. In addition to the relatively small sample sizes of most published studies, the confounding effects of disease-modifying therapies [97] and disease heterogeneity have to be also considered. It is not uncommon that group heterogeneity is further increased by the inclusion of different disease stages [140], phenotypes, and genotypes in an effort to boost sample sizes. Often only a few parameters are evaluated, such as eccentricity or cross-sectional area only, and some studies only assess structural metrics without evaluating diffusivity indices. The spinal studies that do include diffusion sequences often only evaluate the corticospinal tracts (CST) and posterior columns, while other spinal tracts such as vestibulospinal, spinocerebellar, rubrospinal, reticlospinal, and tectospinal tracts are somewhat overlooked. Spinocerebellar projections in particular are also surprisingly understudied despite their likely involvement in a range of gait disorders. The cerebellum is not only involved in SCAs, FRDA, and other primary ataxia syndromes but also in HSP [167] and ALS [19,168,169]; therefore, spinocerebellar and efferent cerebellar projections should be evaluated in more detail. Similarly, several neurodegenerative disorders are classically conceptualized as “pure” brain disorders, even though spinal cord involvement has been demonstrated in Parkinson’s disease [144,145], multiple systems atrophy [170], Huntington’s disease [130], and Alzheimer’s disease [171,172], and spinal cord pathology in these conditions is likely to contribute to the heterogeneity of clinical presentations.

Extrapyramidal gait impairment is also increasingly recognized in motor neuron diseases [173,174,175,176,177], therefore the assessment of spinal white matter tracts should be extended beyond the corticospinal tracts to evaluate tracts like the rubrospinal or spinocerebellar tracts. From an image quality perspective, there is scope to improve MR imaging acquisition and resolution via higher MRI field strength [64,178,179], cardiac- and respiratory-gating [97], which is not always implemented. Alternative MR approaches such as neurite orientation dispersion and density imaging (NODDI), qMT (quantitative magnetization transfer (qMT), MRS, quantitative susceptibility mapping (QSM), functional MRI (fMRI), high angular resolution diffusion imaging (HARDI), and q-ball imaging (QBI) are seldom utilized despite the feasibility of applying these techniques to the spinal cord [3,4,5,6,9,180,181,182,183,184,185]. Spinal PET is also in its infancy in neurodegenerative conditions despite promising studies [186], method papers [7,187], established physiological patterns [188], and oncological applications [189]. While a high number of excellent spinal and outstanding brain imaging studies have been published in most neurodegenerative conditions, there is a striking paucity of integrative studies evaluating the functional interplay and structural connectivity between the brain and cord in the same study with simultaneous brain and cord acquisition [190,191,192,193]. Despite the feasibility, the decussation of the corticospinal tracts is seldom studied. While cerebral sexual dimorphism has a considerable literature both on healthy [194,195] and disease populations [33,34], there is limited literature on cord sexual dimorphism, despite anecdotal observations of notable sex differences in cord metrics. Unfortunately, the complementary clinical scales used in some of these studies are often suboptimal as they may not be validated for a specific condition and may not be necessarily representative of cord pathology [53,136,137]; for example, ALSFRS-R has a bulbar component and is not validated in MND subtypes PMA and PLS [37]. The acquisition of data from relevant spinal levels is a major issue, as some conditions preferentially involve the cervical or thoracic cord. Additionally, superior spinal segments are sometimes analyzed from brain acquisitions, but spinal data in these studies are typically limited to C1-C4 segments [39,40]. This is not an ideal approach, and clinically relevant segments innervating the upper limbs are left out of the field of view (FoV). Dedicated spinal acquisitions seem indispensable. Finally, there are limitations that are specific to longitudinal studies. There may be a selection bias towards less disabled patients, as patients with more severe disability may not be able to participate in follow-up assessments. Some MRI metrics may exhibit “flooring“ and “ceiling“ effects, i.e., patients with long-standing disease may already have maximal spinal cord atrophy or diffusivity change at initial assessment [100]. Follow-up intervals vary significantly in published studies but are often regarded as too short to capture significant radiological changes in slowly progressive neurodegenerative disorders [97]. Similar to early brain imaging studies, the vast majority of quantitative spinal studies compare a specific disease cohort to healthy controls [113] instead of contrasting that cohort to relevant disease controls or other patient groups. The main problem with this approach is that the specificity of the identified changes to that condition remains unclear. Studies contrasting several syndromes [63] and establishing distinguishing imaging features seem superior in this regard and foretell future clinical applications. For example, both PLS and ALS are associated with considerable spinal cord involvement [77,78,196]; therefore, contrasting them individually to healthy controls is of limited relevance. However, the identification of distinguishing spinal features between the two conditions would be hugely helpful, as the two conditions have very different survival prospects and can sometimes be difficult to distinguish clinically in the early phase of the disease [75,196]. In an ideal study, patients with HSP, SBMA, PLS, ALS, and SCA would undergo spinal imaging with a standardized protocol so that the distinguishing imaging signatures could be ascertained. This would translate into genuine clinical utility for the interpretation of an individual scan. Unlike in brain imaging, the absence of large multi-cohort spinal studies precludes the development and validation of machine-learning protocols. Reminiscent of the early days of quantitative brain imaging, most of the published spinal studies are single-center initiatives contributing to the small sample sizes. The lessons and achievements of brain imaging, such as data sharing, standardized protocols, and disease-specific consortia such as the Alzheimer’s Disease Neuroimaging Initiative (ADNI), the Canadian ALS Neuroimaging Consortium (CALSNIC), the Neuroimaging Society in ALS (NisALS), and the Genetic Frontotemporal Initiative (GENFI) [197], should be urgently taken into account to establish spinal data repositories, encourage knowledge and method exchange, and foster cross-site cross-border collaborations. Despite pioneering spinal papers [47,67,96,103,107] and ample examples from brain imaging [70], pre-symptomatic spinal imaging has a strikingly limited literature despite the fact that many motor neuron and neurodegenerative diseases are genetic (HSP, SCA, SOD1-ALS, C9orf72-ALS, etc.) With very few exceptions [140], in the absence of complementary post-mortem data, the histopathological correlates of in vivo spinal signal alterations are not described. This is an important shortcoming, as other than insights from animal studies [1,2], the specific histological correlates of radiological changes are not established. In an ideal study design, the ante-mortem radiological spinal changes should be mapped to post-mortem changes to establish whether a certain radiological WM marker primarily reflects axonal or myelin-related change and also to evaluate the ante-mortem detection sensitivity of specific radiological markers. The candid discussion of stereotyped study limitations may help to improve the design of future spinal studies and enable the development of viable clinical tools.

## 11. Research Priorities for the Development of Viable Clinical Tools

Descriptive, single-cohort academic studies should be superseded by clinically oriented studies focusing on the development of viable diagnostic, prognostic, and monitoring protocols (Table 6). A key focus of forthcoming spinal studies should be the development of single-subject data interpretation frameworks where the quantitative data from a specific individual could be accurately classified into clinically relevant diagnostic, phenotypic, and prognostic categories. Spinal tracts beyond the descending CST and posterior columns should be evaluated. Olivospinal tracts have been assessed in MSA [170], but vestibulospinal, rubrospinal, reticlospinal, and tectospinal tracts should also be assessed, especially with the advent of novel white matter imaging techniques (HARDI, NODDI, QBI, etc.) and the increasing availability of ultra-high filed strength platforms. In light of pioneering studies [130,144,145,170,171,172], spinal involvement should be comprehensively characterized in neurodegenerative conditions classically considered as “brain diseases” such as PD, MSA, AD, and HD. Where possible, clinical spinal protocols should incorporate 3D sequences without slice gaps to permit post hoc quantitative analyses. In the absence of quantitative analyses, high-quality 3D data, or z-scoring to normative data, the usage of subjective terminology based on visual inspection such as “volume loss”, “thinning”, and “atrophic” should be avoided in standard clinical reporting if possible and quantitative protocols suggested instead. Cardiac and respiratory gating should be routinely considered to correct for physiological noise if relatively longer acquisition times are acceptable. Large openly accessible normative data repositories with corresponding height, age, sex, weight, and occupation data would be of considerable value to interpret single patient data at various centers. The lessons of large disease-specific brain data repositories such as ADNI, GENFI, NiSALS, and Track-HD should be considered for the collection, harmonization, and accessibility of multi-site spinal cord data [198,199,200]. The potential advantages and disadvantages of implementing HARDI, QBI, MRT, fMRI, and PET in cord protocols should be formally assessed to inform the design of future academic studies and establish their potential clinical role beyond their mere feasibility. In light of the success of large pre-symptomatic brain studies in a range of genetic neurodegenerative conditions, more effort should be put into pre-symptomatic spinal imaging to clarify propagation patterns and establish whether some of the detected alterations may indeed be developmental as opposed to neurodegenerative. While the performance of specific imaging modalities has been demonstrated in multiple conditions, real-life clinical translation is awaited. Robust validation frameworks are required for the development of real-world clinical applications. Improved performance of spinal imaging markers may curtail the diagnostic journey of patients with rare neurodegenerative conditions, help to distinguish specific phenotypes within disease continua, and act as objective quantitative monitoring markers in clinical trials, translating into better clinical outcomes. Once new spinal protocols are convincingly validated, utilization can be carried out by either a commercial entity, software, a cloud-based application, or a physician in a hospital.

## 12. Conclusions

The reviewed academic studies not only highlight the feasibility of quantitatively interpreting spinal imaging data but also viable clinical applications. While computational spinal imaging seems to lag behind cerebral imaging, there is a range of promising developments that will no doubt expedite the clinical applications of computational spinal imaging. These include the increasing availability of high-field and ultra-high-filed platforms, relentless advances in machine learning, effective multi-site and international collaborations, reducing the price of genetic testing, increasing accessibility of cloud computing, the establishment of the legal framework for patient data protocols, the availability of a multitude of open-source image analysis suites, the increase in disease-specific imaging consortia, and in general, an increasing awareness of the tangible benefits of quantitative imaging instead of visual image interpretation. These factors, coupled with relentless technological advances, routine genetic testing, and an ever-increasing number of clinical trials in neurodegeneration, suggest that quantitative spinal imaging does not only have a potential clinical role but may soon be integrated in clinical decision making.

## Figures and Tables

**Table 1:** Quantitative spinal cord imaging studies in MND phenotypes.

Year	Authors	Participants	Symptom Duration	Study Design	Follow-Up	Field Strength	MRI Technique	MRI Localization	Cardiac-Gating	Respiratory-Gating	Post-Mortem Data	Genetics	Study Outcome	Summary of Quantitative Spinal Cord Imaging Results
**ALS**														
2009	Agosta	ALS *n* = 17 Controls *n* = 20	Median (range): 27 (12–58) months	Longitudinal; case control	Mean 9 (6–12) months	1.5 T	Spinal cord area DTI T2 hyperintensities	Cervical	No	No	No	N/A	Positive	In ALS, there was progressive cord atrophy and structural changes (FA and MD). This was independent of brain changes.
2018	Agosta	SOD1 ALS *n* = 20; sporadic ALS *n* = 11	Mean ± SD: SOD1 ALS 69.9 ± 50.5; sporadic ALS 27.9 ± 29.0 months	Cross-sectional; case control	N/A	1.5 T	Spinal cord area MTR	Cervical	No	No	No	SOD1 ALS *n* = 20	Positive	In SOD1 ALS, there was significant cervical cord atrophy compared with sporadic ALS. Cervical cord atrophy correlated with disease duration and functional impairment in both SOD1 and sporadic ALS
2022	Barry	ALS *n* = 15 Controls *n* = 17	13.2 (8.0) months	Cross-sectional; case control	N/A	3.0 T	Spinal cord area	Cervical Thoracic Lumbar	No	No	No	C9orf72 *n* = 1; SOD1 *n* = 1; TBK1 *n* = 1; Unknown *n* = 12	Positive	In ALS, there was cervical, but not thoracolumbar, cord atrophy.
2014	Branco	ALS *n* = 43 Controls *n* = 43	Mean ± SD: 34.0 ± 29.9 months	Cross-sectional; case control	N/A	3.0 T	Spinal cord area	Cervical	No	No	No	N/A	Positive	In ALS, there was cervical cord atrophy without flattening compared to controls. The degree of atrophy correlated with disease duration and severity.
2016	Budrewicz	ALS *n* = 15 Controls *n* = 15	Mean: 7.3 months	Cross-sectional; case control	N/A	1.5 T	DTI	Cervical	No	No	No	N/A	Positive	In ALS, there were decreased FA values in the cervical cord with a caudal predominance in the anterior, lateral, posterior columns and central cord.
2011	Carew	ALS *n* = 14 Controls *n* = 16	Median (IQR): 26.7 (16.8–36.3)	Cross-sectional; case control	N/A	3.0 T	MRS	Cervical	No	No	No	N/A	Positive	In ALS, there was reduced NAA/Cr, NAA/mIns, Cho/Cr ratios in the cervical cord compared with controls. The reduced NAA/mIns and NAA/Cho ratios correlated with worsening respiratory function. The neurometabolite ratios did not correlate with clinical disability.
2011	Carew	Pre-symptomatic SOD1 *n* = 24; SOD1 ALS *n* = 23 Controls *n* = 29	SOD1 ALS Mean (IQR): 567 (262.5–1084) days	Cross-sectional; case control	N/A	3.0 T	MRS	Cervical	No	No	No	Pre-symptomatic SOD1 *n* = 24; ALS SOD1 *n* = 23	Positive	In pre-symptomatic SOD1, the altered metabolite ratios (reduced NAA/Cr, NAA/mIns and mIns/Cr) in the cervical cord were similar to symptomatic SOD1 ALS (reduced NAA/Cr, NAA/mIns and NAA/Cho) compared with controls. This suggests that metabolic changes occur in early disease.
2013	Cohen-Adad	ALS *n* = 29 Controls *n* = 21	Mean 1.4 years	Cross-sectional; Case control	N/A	3.0 T	Spinal cord area DTI MTR	Cervical	Yes	No	No	SOD1 *n* = 2 Sporadic *n* = 27	Positive	In ALS, there was cervical cord atrophy; reduced FA and increased RD in the lateral CST and dorsal regions; and reduced MTR in the lateral CST of the cervical cord. Reduced FA of lateral CST correlated with clinical severity. Focal spinal cord atrophy was associated with corresponding muscle weakness.
2013	Cohen-Adad	ALS *n* = 1 Controls *n* = 1	23 months	Cross-sectional; case control	N/A	7.0 T	T2 hyperintensities	Cervical	No	No	No	N/A	Positive	7.0T MRI detected T2 hyperintensities in the lateral CST of the cervical cord in a single patient with ALS compared to a control
2017	deAlbuquerque	ALS *n* = 27 Controls *n* = 27	Median (range): 30.5 (16–150) months	Longitudinal; case control	8 months	3.0 T	Spinal cord area	Cervical	No	No	No	C9orf72 negative ALS *n* = 27	Positive	In sporadic ALS, there was progressive cervical spinal cord atrophy without flattening that correlated with disease severity (ALSFRS-R).
2014	ElMendili	ALS *n* = 29, baseline ALS *n* = 14, follow-up	26.8 ± 26.9 months	Longitudinal; case series	11 ± 3 months	3.0 T	Spinal cord area DTI MTR	Cervical	Yes	No	No	N/A	Positive	In ALS, there was longitudinal cervical cord atrophy and decreased MTR. The rate of atrophy correlated with upper limb ALSFRS-R and MMT scores.
2018	Fukui	ALS *n* = 38; HSP *n* = 7; SBMA *n* = 6 Controls *n* = 8	ALS: 1.3 ± 1; SBMA: 16.5 ± 7; HSP: 15.3 ± 7.7 years	Cross-sectional; case control	N/A	3.0 T	DTI	Cervical	No	No	No	SBMA *n* = 6	Positive	In ALS, there was reduced FA in the cervical cord compared with controls and SBMA. The rate of ALS disease progression of significantly correlated with FA.
2018	Grolez	ALS *n* = 40 Controls *n* = 21	N/A	Longitudinal; case control	3 months	3.0 T	Volumetry	Cervical	No	No	No	N/A	Positive	In ALS, the longitudinal decrease in cervical cord volume over 3 months was predictive of the change in slow vital capacity at 12 months.
2015	Iglesias	ALS *n* = 21 Controls *n* = 21	26.6 ±3.6 months	Cross-sectional; case control	N/A	3.0 T	DTI	Cervical	Yes	No	No	N/A	Positive	In ALS, there was increased RD and MD in the posterior columns; and increased RD, MD and reduced FA in the lateral CST of the cervical cord. No correlation of DTI metrics with clinical measures.
2013	Ikeda	ALS *n* = 19 Controls *n* = 20	Mean ± SD: 20.4 ± 8.0 months	Cross-sectional; case control	N/A	1.5 T	MRS	Cervical	No	No	No	N/A	Positive	In ALS, there was reduced NAA/Cr, NAA/mIns and increased mIns/Cr ratios in the cervical cord compared with controls. NAA/Cr and NAA/mIns ratios were significantly associated with ALSFRS and FVC and inversely linked to annual decline in ALSFRS and FVC.
2022	Leoni	Pre-symptomatic VAPB *n* = 10; VAPB ALS *n* = 20; Sporadic ALS *n* = 20; Controls *n* = 30	Mean ± SD: 6.4 ± 4.4 years	Cross-sectional; case control	N/A	3.0 T	Spinal cord area	Cervical	No	No	No	Pre-symptomatic and symptomatic VAPB ALS *n* = 30; sporadic ALS (C9orf72, ATXN2, VAPB, SOD1 negative) *n* = 20	Positive	There is cervical spinal cord atrophy in pre-symptomatic and symptomatic VAPB-related ALS. The degree of atrophy was greater in the symptomatic VAPB-related ALS. This did not correlate with disease duration or ALSFRS.
2010	Nair	ALS *n* = 14 Controls *n* = 15	Mean (IQR): 2 (1.1–3.8) years	Cross-sectional; case control	N/A	3.0 T	DTI	Cervical	No	No	No	N/A	Positive	In ALS, there was reduced FA and increased RD in the cervical cord—more prominent in the distal cervical cord—compared with controls. Reduced FA correlated with average finger and foot tapping speed; and increased RA correlated with average finger and foot tapping speed, ALSFRS-R, and FVC
2023	Nigri	ALS *n* = 48 Controls *n* = 17	Median (IQR): 14 (9–27) months	Cross-sectional; case control	N/A	3.0 T	Spinal cord area	Cervical	No	No	No	Sporadic ALS *n* = 48 negative for C9orf72, SOD1, FUS, OPTN, TARDBP	Positive	In ALS, cervical cord atrophy spreads from GM to WM across the clinical King’s Staging System. Cervical GM and WM atrophy correlates with disability.
2018	Olney	ALS *n* = 10 Controls *n* = 10	Mean (range): 44 (11–128) months	Cross-sectional; case control	N/A	3.0 T	Spinal cord area	Cervical	No	No	No	N/A	Positive	In ALS, there reduced total cervical cord CSA, GM, and WM area.
2018	Paquin	ALS *n* = 29 Controls *n* = 22	N/A	Cross-sectional; case control	N/A	3.0 T	Spinal cord area	Cervical	No	No	No	SOD1 *n* = 2	Positive	Cervical cord GM atrophy is more sensitive than cross-sectional atrophy at differentiating patients with ALS from controls. Cervical cord GM and cross-sectional area correlated with baseline and 1-year clinical disability scores. ALSFRS-R prediction at 1 year improves with combined GM and WM area.
2019	Patzig	ALS *n* = 14 Controls *n* = 15	Median (range): 20 (5–108) months	Cross-sectional; case control	N/A	3.0 T	DTI	Cervical	No	No	No	N/A	Positive	In ALS, there was reduced CSA at C2-C4 and reduced FA in the cervical cord anterolateral motor tracts compared with controls. These findings did not correlate with clinical measures.
2020	Pisharady	ALS *n* = 20 Controls *n* = 20	38.5 ± 42.5 (3.4–147.7) months	Longitudinal; case control	6 months (*n* = 10); 12 months (*n* = 11)	3.0 T	Spinal cord area DTI	Cervical	No	No	No	N/A	Positive	In ALS, there was reduced cervical cord CSA; reduced FA in the lateral CST; and a trend towards reduced FA in the posterior columns. There was longitudinal increase in RD and MD. Multimodal analysis of both cervical cord CSA and FA significantly improved sensitivity in differentiating from patients with ALS from controls. There was a strong correlation between CSA, FA, and ALSFRS-R scores.
2018	Querin	ALS *n* = 60 Controls *n* = 45	Mean ± SD (range): 30 ± 30 (1–168) months	Cross-sectional; case control	N/A	3.0 T	Spinal cord area DTI MTR	Cervical	Yes	No	No	N/A	Positive	A classification model using cervical cord CSA, DTI and MTR variables differentiated ALS from controls (sensitivity 88%; specificity 85%; AUC 0.96). The best-performing individual variables were RD, FA, CSA at C5 spinal level.
2017	Querin	ALS *n* = 49	Mean ± SD: 27.9 ± 28.52 months	Cross-sectional; case series	N/A	3.0 T	Spinal cord area DTI MTR	Cervical	Yes	No	No	N/A	Positive	In ALS, reduced CSA, FA and MTR in cervical cord are all associated shorter disease duration. Multi-modal cord analyses could predict survival in ALS.
2019	Querin	Pre-symptomatic C9orf72 *n* = 40 Controls *n* = 32	N/A	Longitudinal; case control	18 months	3.0 T	Spinal cord area DTI	Cervical	Yes	No	No	C9orf72 *n* = 40	Positive	In pre-symptomatic C9orf72 aged >40 years, there was cervical cord WM atrophy at baseline; and reduced FA in cervical CST at longitudinal follow-up.
2017	Rasoanandrianina	ALS *n* = 10 Controls *n* = 20	Median (IQR): 15.5 (11–22) months	Cross-sectional; case control	N/A	3.0 T	Spinal cord area DTI MTR ihMTR	Cervical	Yes	No	No	N/A	Positive	In ALS, there was cervical cord GM and WM atrophy, reduced FA in CST; reduced AxD in CST and posterior columns; reduced MTR in CST; and reduced ihMTR in all ROIs. These MRI metrics strongly correlated with clinical scores.
2005	Sperfeld	ALS *n* = 39; LMND *n* = 19; SBMA *n* = 19; Controls *n* = 96	Mean ± SD: ALS: 3 ± 4; SBMA: 24 ± 11; LMND: 21 ± 18 years	Cross-sectional; case control	N/A	1.5 T	T2 hyperintensities AP diameter	Cervical Thoracic	No	No	No	SBMA *n* = 19	Negative	No difference in cervical and thoracic cord thickness or altered signal in CST or posterior columns in ALS compared to controls.
2023	Toh	ALS *n* = 75; Controls *n* = 13	Mean ± SD: 17 ± 17 months	Cross-sectional; case control	N/A	3.0 T	Spinal cord area	Cervical	No	No	No	Case-by-case basis, and not systematically in all patients. C9orf72+ *n* = 1; and SOD1+ *n* = 1	Negative	In ALS, there was no difference in cervical cord CSA compared to controls. There was a positive cranio-caudal step-wise gradient between intracranial CST and C1, and between cervical cord C5 and C6 levels.
2007	Valsasina	ALS *n* = 28; Controls *n* = 20	Median (range): 26 (6–58) months	Cross-sectional; case control	N/A	1.5 T	Spinal cord area DTI T2 hyperintensities	Cervical	No	No	No	N/A	Positive	In ALS, there was reduced cervical cord CSA and FA. FA correlated with ALSFRS.
2019	vanderBurgh	(*n* = baseline, follow-up) C9orf72-ALS *n* = 108, 64 C9orf72 + ALS *n* = 26; 18 PLS *n* = 28; 18 PMA *n* = 56; 41 Controls *n* = 114, 54	Median (range): C9orf72-ALS 14.4 (2.9–174.4); C9orf72 + ALS 11.5 (3.3–68.3); PLS 90.6 (23.5–224.9); PMA 20.3 (4.6–467.4) months	Longitudinal; case control	Median (range): C9orf72-ALS 4.9, (3.0–9.8); C9orf72 + ALS 5.4, (3.0–10.0); PLS 6.8 (3.2–85.9); PMA 5.5 (3.0–59.0) months	3.0 T	Spinal cord area	Cervical	No	No	No	C9orf72 + ALS *n* = 26	Positive	In C9orf72 + ALS and C9orf72-ALS, there was upper cervical cord atrophy that was progressive in C9orf72-ALS but not in C9orf72 + ALS. Disease progression measured by longitudinal ALSFRS-R scores correlated with cervical cord atrophy in C9orf72-ALS.
2014	Wang	ALS *n* = 24 Controls *n* = 16	Range: 6–42 months	Cross-sectional; case control	N/A	1.5 T	DTI	Cervical	No	No	No	N/A	Positive	In ALS, there was decreased FA in bilateral lateral CSTs compared to controls. It did not correlate with disease severity.
2020	Wimmer	ALS *n* = 158 (incl. PLS *n* = 9) Controls *n* = 86	Median (range): 16 (3–272) months	Longitudinal; case control	6-months	3.0 T	Spinal cord area	Cervical	No	No	No	ALS *n* = 63 tested for SOD1 and C9orf72 (SOD1+ *n* = 7 and C9orf72+ *n* = 7)	Positive	In ALS, there was cervical cord atrophy, with a trend towards longitudinal progression at 6-month interval. It was most marked in limb-onset ALS. Baseline cervical cord CSA correlated with ALSFRS-R disease severity.
**PLS**														
2019	Dvorak	PLS *n* = 2 RRMS *n* = 1; PPMS *n* = 1; NMO *n* = 2	N/A	Cross-sectional; case control	N/A	3.0 T	DTI MWI	Cervical	No	No	No	N/A	Positive	In PLS, there was low myelin water fraction in the lateral funiculi; high RD in GM; low FA in whole cord, GM and lateral funiculi compared to controls.
2019	vanderBurgh	(*n* = baseline, follow-up) C9orf72-ALS *n* = 108, 64 C9orf72 + ALS *n* = 26; 18 PLS *n* = 28; 18 PMA *n* = 56; 41 Controls *n* = 114, 54	Median (range): C9orf72-ALS 14.4 (2.9–174.4); C9orf72 + ALS 11.5 (3.3–68.3); PLS 90.6 (23.5–224.9); PMA 20.3 (4.6–467.4) months	Longitudinal; case control	Median (range): C9orf72-ALS 4.9, (3.0–9.8); C9orf72 + ALS 5.4, (3.0–10.0); PLS 6.8 (3.2–85.9); PMA 5.5 (3.0–59.0) months	3.0 T	Spinal cord area	Cervical	No	No	No	C9orf72 + ALS *n* = 26	Positive	In PLS, there was upper cervical cord atrophy without longitudinal changes. This correlated with disease progression measured by longitudinal ALSFRS-R.
2020	Wimmer	ALS *n* = 158 (incl. PLS *n* = 9) Controls *n* = 86	Median (range): 16 (3–272) months	Longitudinal; case control	6 months	3.0 T	Spinal cord area	Cervical	No	No	No	ALS *n* = 63 tested for SOD1 and C9orf72 (SOD1+ *n* = 7 and C9orf72+ *n* = 7)	Positive	In ALS, there was cervical cord atrophy, with a trend towards longitudinal progression at 6-month interval. It was most marked in limb-onset ALS. Baseline cervical cord CSA correlated with ALSFRS-R disease severity.
**PMA**														
2005	Sperfeld	ALS *n* = 39; LMND *n* = 19; SBMA *n* = 19; Controls *n* = 96	Mean ± SD: ALS: 3 ± 4; SBMA: 24 ± 11; LMND: 21 ± 18 years	Cross-sectional; case control	N/A	1.5 T	T2 hyperintensities AP diameter	Cervical Thoracic	No	No	No	SBMA *n* = 19	Negative	In PMA, there was no difference in cervical and thoracic spinal cord thickness and no signal alterations in CST or posterior columns compared to controls.
2019	vanderBurgh	(*n* = baseline, follow-up) C9orf72-ALS *n* = 108, 64 C9orf72 + ALS *n* = 26; 18 PLS *n* = 28; 18 PMA *n* = 56; 41 Controls *n* = 114, 54	Median (Range): C9orf72-ALS 14.4 (2.9–174.4); C9orf72 + ALS 11.5 (3.3–68.3); PLS 90.6 (23.5–224.9); PMA 20.3 (4.6–467.4) months	Longitudinal; case control	Median (range): C9orf72-ALS 4.9, (3.0–9.8); C9orf72 + ALS 5.4, (3.0–10.0); PLS 6.8 (3.2–85.9); PMA 5.5 (3.0–59.0) months	3.0 T	Spinal cord area	Cervical	No	No	No	C9orf72 + ALS *n* = 26	Positive	In PMA, there is progressive upper cervical cord atrophy. Disease progression measured by longitudinal ALSFRS-R scores correlated with atrophy in PMA.
**SBMA**														
2018	Fukui	ALS *n* = 38; HSP *n* = 7; SBMA *n* = 6 Controls *n* = 8	ALS: 1.3 ± 1; SBMA: 16.5 ± 7; HSP: 15.3 ± 7.7 years	Cross-sectional; case control	N/A	3.0 T	DTI	Cervical	No	No	No	SBMA *n* = 6	Negative	In SBMA, there was no difference in DTI metrics compared with controls.
2005	Sperfeld	ALS *n* = 39; LMN disease *n* = 19; SBMA *n* = 19; Controls *n* = 96	Mean ± SD: ALS: 3 ± 4; SBMA: 24 ± 11; LMND: 21 ± 18 years	Cross-sectional; case control	N/A	1.5 T	T2 hyperintensities AP diameter	Cervical Thoracic	No	No	No	SBMA *n* = 19	Positive	In SBMA, there was cervical and thoracic spinal cord atrophy and no signal alterations in CST or posterior columns compared to controls.
**PPS**														
2022	Wendebourg	PPS *n* = 20 Controls *n* = 20	Mean, median (range): 44.4, 43.5 (25–61)	Cross-sectional; case control	N/A	3.0 T	Spinal cord area	Cervical Thoracic	Yes	No	No	N/A	Positive	In PPS, there was cervical and thoracic cord atrophy that correlated with muscle strength in corresponding myotomes. It was associated with patient-reported PPS-related functional decline. The atrophy was more marked in those with progressive upper limb symptoms compared to stable disease.
**SMA**														
2016	ElMendili	SMA *n* = 18 (Type IIIa *n* = 5; IIIb *n* = 10; IV *n* = 3) Controls *n* = 18	26 ± 15 years	Cross-sectional; case control	N/A	3.0 T	Spinal cord area	Cervical	No	No	No	SMN1 SMA Type III or IV *n* = 18	Positive	In adult SMA, there was cervical cord atrophy—particularly in segments that innervate the proximal muscles. There was no correlation with strength or disability scores.
2021	Querin	SMA Type III or IV *n* = 14	N/A	Longitudinal; case series	24 months	3.0 T	Spinal cord area	Cervical	No	No	No	SMA Type III or IV *n* = 14 (SMN2 4 copies *n* = 11; SMN2 3 copies *n* = 3)	Negative	In SMA type III and IV, there was no significant longitudinal difference in cervical cord GM-CSA and WM-CSA between baseline and 2-year scans
2019	Querin	SMA *n* = 25 (Type III *n* = 19; IV *n* = 6) Controls *n* = 25	Mean ± SD (range): 30.38 ± 15.4 (5–55) years	Cross-sectional; case control	N/A	3.0 T	Spinal cord area DTI	Cervical	Yes	No	No	SMN1 SMA Type III or IV *n* = 25	Positive	In SMA type III and IV, there was whole cervical cord atrophy with selective GM degeneration. GM-CSA at C3-C4 correlated with deltoid strength.
2019	Stam	SMA *n* = 10 (Type II *n* = 1; IIIa *n* = 4; IIIb *n* = 5) Controls *n* = 30	N/A	Cross-sectional; case control	N/A	3.0 T	Spinal cord area DTI	Cervical	No	No	No	SMA Type II or III *n* = 10	Positive	In SMA, there was increased AxD in the cervical GM; reduced MD, AxD and RD in the cervical nerve roots; and a trend towards whole cervical cord atrophy, greatest at C7 level. These imaging findings did not correlate with clinical measures.

**Table 2:** Quantitative spinal cord imaging studies in hereditary ataxias.

Year	Authors	Participants	Symptom Duration	Study Design	Follow-Up	Field Strength	MRI Technique	MRI Localization	Cardiac-Gating	Respiratory-Gating	Post-Mortem Data	Genetics	Study Outcome	Summary of Quantitative Spinal Cord Imaging Results
**SCA**														
2021	Faber	SCA3 Ataxic *n* = 210 SCA3 pre-ataxic *n* = 48 Controls *n* = 63	Mean (SD): SCA3 Ataxic 13 ± 9; SCA3 pre-ataxic (estimated) -2 ± 9 years	Cross-sectional; case control	N/A	Mixed	Spinal cord area	Cervical	No	No	No	SCA3 Ataxic *n* = 210; SCA3 Pre-ataxic *n* = 48	Positive	In SCA3, there is cervical cord atrophy before symptomatic ataxia. There was reduced cervical cord CSA in ataxic SCA3 compared with pre-ataxic SCA3 and in pre-ataxic SCA3 compared with controls.
2015	Fahl	SCA3 *n* = 48 Controls *n* = 48	Mean (SD: 9 ± 4 years	Cross-sectional; case control	N/A	3.0 T	Spinal cord area	Cervical	No	No	No	SCA3 *n* = 48	Positive	In SCA3, there was cervical cord atrophy and anteroposterior flattening compared with controls. The cervical cord CSA correlated with ataxia severity and disease duration.
2021	Hernandez-Castillo	SCA7 *n* = 48 Controls *n* = 48	Mean (SD): 9.5 (6.3) years	Cross-sectional; case control	N/A	3.0 T	Spinal cord area	Cervical	No	No	No	SCA7 *n* = 48	Positive	In SCA7, there was cervical cord atrophy and increased eccentricity. The degree of cervical cord atrophy correlated with disease severity measured by SARA and was associated with disease duration.
1996	Higgins	AD SCA *n* = 34 Controls = not specified	N/A	Longitudinal; case control	1 year	0.5T	Spinal cord area	Cervical	No	No	No	SCA1n = 7 SCA3n = 17 Not SCA1 or 3 *n* = 10	Positive	In SCA3 and SCA1, there was reduced cervical cord CSA, without longitudinal progression at 1 year.
2008	Lukas	SCA3 *n* = 14 SCA6 *n* = 10 Controls *n* = 24	SCA3 *n* = 14 Mean 7 years SCA6 *n* = 10 Mean 9 years	Cross-sectional; case control	N/A	1.5 T	Spinal cord area	Cervical	No	No	No	SCA3 *n* = 14; SCA6 *n* = 10	Positive	In SCA3, there was cervical cord atrophy that did not correlate with clinical severity. In SCA6, there was normal cervical cord CSA, but lower mean cervical cord CSAs correlated with clinical severity.
2017	Martins Jr	SCA1 *n* = 31 Controls *n* = 31	Mean (SD): 7.7 ± 6.5 years	Cross-sectional; case control	N/A	3.0 T	Spinal cord area	Cervical	No	No	No	SCA1 *n* = 31	Positive	In SCA1, there was cervical spinal cord atrophy and flattening. The degree of cervical cord atrophy correlated with disease severity and was associated with disease duration.
2020	Piccinin	SCA3 *n* = 23 Controls *n* = 22	Mean (SD): 9 ± 5 years	Longitudinal; case control	5 years	3.0 T	Spinal cord area	Cervical	No	No	No	SCA3 *n* = 23	Negative	In SCA3, there was no longitudinal change in cervical cord CSA over 5 years. This may be because the mean disease duration is 9 years and maximal spinal cord atrophy has already occurred.
2018	Rezende	SCA3 *n* = 79 Pre-symptomatic SCA3 *n* = 12 Controls *n* = 91	Mean (SD) 10 ± 7 years	Cross-sectional; case control	N/A	3.0 T	Spinal cord area	Cervical	No	No	No	SCA3 *n* = 79 pre-symptomatic SCA3 *n* = 12	Positive	There was reduced cervical cord CSA in all groups including pre-symptomatic and symptomatic SCA3 (stratified: <5years, 5–10 years, 10–15 years and >15 years), with linear progression over time.
**FDRA**														
2013	Chevis	FRDA *n* = 33 Controls *n* = 30	10.6 ± 8.3 years	Cross-sectional; case control	N/A	3.0 T	Spinal cord area	Cervical	No	No	No	FRDA *n* = 33	Positive	In FDRA, there is cervical cord atrophy and flattening compared with controls. The cervical cord CSA correlates with disease severity as measured by FARS.
2019	Dogan	FDRA *n* = 21 Controls *n* = 22	Mean ± SD: 18.95 ± 9.08 years	Cross-sectional; case control	N/A	3.0 T	Spinal cord area Volumetry	Cervical Thoracic	No	No	No	FRDA *n* = 21	Positive	In FRDA, there was reduced CSA, volume and flattening of the cervical and thoracic cord. There was greater atrophy in the cervical cord, and greater flattening in the distal thoracic cord. CSA and volume were associated with disease severity.
2022	Hernandez	FDRA *n* = 30 Controls *n* = 30	11 ± 9 years	Cross-sectional; case control	N/A	3.0 T	Spinal cord area DTI	Cervical	No	No	No	FDRA *n* = 30	Positive	In FDRA, there is cervical cord atrophy and flattening. The DTI abnormalities in the total WM, dorsal columns, fasciculus gracilis, fasciculus cuneatus, and CSTs in all cervical levels correlated with disease duration and severity.
2022	Joers	FDRA *n* = 28 Controls *n* = 20	Mean ± SD: 5.5 ± 4.0 years	Longitudinal; cross-sectional; case control	1 year *n* = 21; 2 year *n* = 19	3.0 T	Spinal cord area DTI MRS	Cervical	Yes	No	No	FDRA *n* = 28	Positive	In FDRA at baseline, there was reduced cervical cord CSA and higher eccentricity; reduced FA, increased RD, MD and AxD; and reduced tNAA/mIns ratio compared to controls. At follow-up, there was progressively reduced cervical cord CSA; reduced tNAA/mIns ratio; and a trend towards decreasing in FA. Cervical cord CSA correlated with disease severity (SARA, FARS). FA and tNAA/mIns also correlated with clinical measures, but to a lesser extent.
2018	Rezende	FDRA *n* = 38 (Adult FDRA *n* = 25; Young FDRA *n* = 12) Controls *n* = 37	Mean ± SD (range): Adult FDRA: 15 ± 11 (4–46); Young FDRA 6 ± 3 (2–9) years	Cross-sectional; case control	N/A	3.0 T	Spinal cord area	Cervical	No	No	No	FDRA *n* = 38	Positive	In both adult- and young-onset FDRA, there was cervical cord atrophy and increased eccentricity. In young FDRA, cervical cord CSA correlated with age.
2023	Rezende	FDRA *n* = 256 Controls *n* = 223	Mean ± SD: 14 ± 10 years	Cross-sectional; case control	N/A	3.0 T	Spinal cord area	Cervical	No	No	No	FDRA *n* = 256	Positive	In FDRA, there was reduced cervical cord CSA that correlated with disease severity; and increased eccentricity that did not correlate with clinical metrics.
**ACRA**														
2018	Gama	SYNE1 *n* = 6 Controls *n* = 6	Mean 10.2 ± 2.6 years	Cross-sectional; case control	N/A1	3.0 T	Spinal cord area	Cervical	No	No	No	SYNE1 *n* = 6	Negative	In SYNE-ataxia, there was no significant cervical spinal cord involvement

**Table 3:** Quantitative spinal cord imaging studies in HSP.

Year	Authors	Participants	Symptom Duration	Study Design	Follow-Up	Field Strength	MRI Technique	MRI Localization	Cardiac-Gating	Respiratory-Gating	Post-Mortem Data	Genetics	Study Outcome	Summary of Quantitative Spinal Cord Imaging Results
2015	Agosta	Pure HSP *n* = 20; complicated HSP *n* = 24 Controls *n* = 19	Mean ± SD (range): pHSP 26.1 ± 13.1 (8–47); cHSP 18.1 ± 9.7 (4–51) years	Cross-sectional; case control	N/A	1.5 T	Spinal cord area	Cervical	No	No	No	SPG4 *n* = 11; SPG11 *n* = 3; SPG15 *n* = 2; SPG3A *n* = 1; SPG5 *n* = 1; SPG7 *n* = 1; SPG10 *n* = 1	Positive	In pHSP and cHSP, there was upper cervical cord atrophy compared with controls. No difference in cord atrophy between pHSP and cHSP. No correlation between cord atrophy and disease severity.
2018	Faber	SPG11 *n* = 25 Controls *n* = 25	Mean (range): 13.2 (0–30) years	Cross-sectional; case control	N/A	3.0 T	Spinal cord area	Cervical	No	No	No	SPG11 *n* = 25	Positive	In SPG11, there was reduced cervical cord CSA without changes in eccentricity. The cervical cord CSA was reduced in patients with longer disease duration and inversely correlated with SPRS.
2018	Fukui	HSP *n* = 7 SBMA *n* = 6 ALS *n* = 38 Controls *n* = 8	HSP: 15.3 ± 7.7 SBMA: 16.5 ± 7; ALS 1.3 ± 1 years	Cross-sectional; case control	N/A	3.0 T	DTI	Cervical	No	No	No	SBMA *n* = 6	Negative	In HSP, no cervical cord DTI abnormalities were detected.
2005	Hedera	HSP *n* = 13 (SPG4 *n* = 5; SPG3A *n* = 3; SPG8 *n* = 3; SPG6 *n* = 2) Controls *n* = 38	Mean (range): 22.2 (7–50) years	Cross-sectional; case control	N/A	1.5 T	Spinal cord area	Cervical Thoracic	No	No	No	SPG4 *n* = 5; SPG3A *n* = 3; SPG8 *n* = 3; SPG6 *n* = 2	Positive	In HSP, there was reduced cervical and thoracic cord CSA compared to controls. It was most marked in SPG6 and SPG8. No correlation between cord atrophy and disease severity in any HSP genotype.
2022	Hocquel	SPG3A *n* = 5 Controls *n* = 8	Mean (range): 25.6 (6–48) years	Cross-sectional; case control	N/A	3.0 T	Spinal cord area Volumetry	Cervical Thoracic	No	No	No	SPG3A *n* = 5	Positive	In SPG3A, significant decrease in cervical and thoracic cord CSA compared to controls.
1997	Krabbe	AD pure HSP *n* = 16 Controls *n* = 8	Range: (4–31) years	Cross-sectional; case control	N/A	1.0 T	Spinal cord area	Cervical Thoracic	No	No	No	N/A	Positive	In pHSP, there was significantly smaller AP diameter of spinal cord at T3 and T9 compared to controls.
2022	Lindig	HSP *n* = 40 (SPG7 *n* = 15; SPG4 *n* = 12; SPG5 *n* = 4; SPG11 *n* = 1) Controls *n* = 125	Median (IQR): 16.5 ± 12 years	Cross-sectional; case control	N/A	3.0 T	DTI	Cervical	Yes	No	No	SPG7 *n* = 15; SPG4 *n* = 12; SPG5 *n* = 4; SPG11 *n* = 1	Positive	In HSP, there was reduced FA, MD and increased RD in the pyramidal tracts; and reduced FA in dorsal columns. No significant correlation between DTI metrics and in all participants. In a subgroup of SPG4 genotype, DTI metric RD in dorsal columns significantly correlated with disease severity.
2022	Liu	SPG5 *n* = 17 Controls *n* = 17	Mean (range): 18 (6–37)	Cross-sectional; case control	N/A	3.0 T	DTI	Cervical Thoracic	No	No	No	SPG5 *n* = 17	Positive	In SPG5, there was WM degeneration in the cervical and thoracic spinal cord compared with controls. It did not correlate with clinical data.
2022	Navas-Sanchez	SPG4 *n* = 12 Controls *n* = 14	Mean: 21.8 years	Cross-sectional; case control	N/A	1.5 T	Spinal cord area DTI	Cervical Thoracic	No	No	No	SPG4 *n* = 12	Positive	In SPG4, there was mild atrophy of the cervical and thoracic spinal cord and tract-specific axonal damage compared with controls. The severity of motor disability negatively correlated with FA, particularly lateral funiculi in cervical cord and dorsal columns in thoracic cord.
2021	Qianqian	SPG5 *n* = 17 Controls = not specified	Median (range): 14 (6–40) years	Cross-sectional; case control	N/A	3.0 T	Spinal cord area	Cervical Thoracic	No	No	No	SPG5 *n* = 17	Positive	In SPG5, there is cervical and thoracic cord atrophy, that was most marked at thoracic levels.
2014	Rezende	SPG4 *n* = 11 Controls *n* = 23	Mean (range): 13.9 (4–30) years	Cross-sectional; case control	N/A	3.0 T	Spinal cord area	Cervical	No	No	No	SPG4 *n* = 11	Positive	In SPG4, there was cervical spinal cord atrophy without flattening compared with controls.
2021	Servelhere	HSP *n* = 37 (SPG3A *n* = 7; SPG4 *n* = 12; SPG7 *n* = 10; SPG11 *n* = 8) Controls *n* = 21	Mean (range): 22.4 years; (SPG3A = 33.0; SPG4 = 21.0; SPG7 = 27.0; SPG11 = 10.0)	Cross-sectional; case control	N/A	3.0 T	Spinal cord area	Cervical	No	No	No	SPG3A *n* = 7; SPG4 *n* = 12; SPG7 *n* = 10; SPG11 *n* = 8	Positive	In SPG4, there was GM and WM cervical cord atrophy; and the GM cervical cord atrophy negatively correlated with disease duration and severity. In SPG11, there was GM and WM cervical cord atrophy; and there were no clinical-imaging correlations. There was no cervical cord atrophy in SP3A or SPG7.
2005	Sperfeld	HSP *n* = 30 (pHSP *n* = 20; cHSP *n* = 10) Controls *n* = 54	pHSP: 19.7 ± 13.1; cHSP 14.9 ± 10.1 years	Cross-sectional; case control	N/A	1.5 T	Spinal cord area	Cervical Thoracic	No	No	No	SPG4 *n* = 6 in pHSP group	Positive	In HSP (both pHSP and cHSP), there was significant cervical and thoracic cord atrophy compared to controls. There was no difference in cervical cord atrophy between pHSP and cHSP. There radiological findings did not correlate with spasticity as measured by Modified Ashworth Scale.

**Table 4:** Quantitative spinal cord imaging studies in other genetic neurodegenerative disorders.

Year	Authors	Participants	Symptom Duration	Study Design	Follow-Up	Field Strength	MRI Technique	MRI Localization	Cardiac-Gating	Respiratory-Gating	Post-Mortem Data	Genetics	Study Outcome	Summary of Quantitative Spinal Cord Imaging Results
**HD**														
2014	Muhlau	HD *n* = 51 Alzheimer’s disease *n* = 35 Controls *n* = 227	N/A	Cross-sectional; case control	N/A	Mixed 1.5 T, 3.0 T	Spinal cord area	Cervical	No	No	No	HD *n* = 51	Positive	In HD, there is cervical spinal cord atrophy that correlates with motor deficits.
2017	Wilhelms	HD *n* = 17 Pres-ymptomatic HD *n* = 27	N/A	Cross-sectional; longitudinal; case control	23 months for pre-symptomatic HD	1.5 T	Spinal cord area	Cervical	No	No	No	HD *n* = 44	Positive	The mean upper cervical cord area was significantly reduced in HD and to a lesser extent in pre-symptomatic HD compared with controls. There was longitudinal progression of cervical cord atrophy in pre-symptomatic HD. It did not correlate with clinical scores related to motor function.
**ALD**														
2016	Castellano	ALD *n* = 13 Controls *n* = 13	10.5 ± 4.3 years	Cross-sectional; case control	N/A	3.0 T	Spinal cord area DTI	Cervical; thoracic	No	No	No	ALD *n* = 13	Positive	In ALD, there was reduced cervical and thoracic cord area compared with controls. It was more marked in the thoracic regions. There was reduced FA, AxD and increased RD in the upper cervical cord. Total cord area and DTI parameters did not correlate with clinical scores.
2019	Politi	ALD *n* = 6 Controls *n* = 6	N/A	Longitudinal; case control	Mean 22.6 months (range 15–29)	3.0 T	Spinal cord area DTI	Cervical; thoracic	No	No	No	ALD *n* = 6	Positive	There was reduced cervical and thoracic spinal cord area in ALD at baseline and at follow-up compared to controls. Progressive atrophy in the upper thoracic cord, and a trend towards progression in lower cervical cord. Reduced WM FA in the upper cervical cord at baseline and at follow-up. Increased MD and RD at C2-C3 levels on follow-up. No significant change in clinical scores at follow-up.
2020	vandeStadt	ALD *n* = 42 Controls *n* = 32	Median (IQR) 15.0 (8–21) years	Cross-sectional; longitudinal; case control	1 year, *n* = 26	3.0 T	Spinal cord area	Cervical	No	No	No	ALD *n* = 42	Positive	In ALD, there was reduced cervical cord CSA and increased eccentricity. The cervical cord CSA correlated with disease severity. No longitudinal change in cervical cord CSA at 1-year follow-up.

**Table 5:** Quantitative spinal cord imaging studies in acquired spinal cord disorders.

Year	Authors	Participants	Symptom Duration	Study Design	Follow-Up	Field Strength	MRI Technique	MRI Localization	Cardiac-Gating	Respiratory-Gating	Post-Mortem Data	Genetics	Study Outcome	Summary of Quantitative Spinal Cord Imaging Results
**HTLV-1**														
2014	Evangelou	Definite HAM/TSP *n* = 5 Possible HAM/TSP *n* = 2 Asymptomatic HTLV1 *n* = 2 Controls *n* = 5	Mean (range) 8.2 (2–13) years	Cross-sectional; case control	N/A	1.5 T	Volumetry	Cervical; thoracic	No	No	No	N/A	Positive	Definite HAM/TSP had reduced cervical and thoracic cord volume compared to controls. Possible HAM/TSP had lower thoracic cord volumes similar to definite HAM/TSP. Asymptomatic HTLV1 had thoracic cord volumes similar to controls. Imaging metrics did not correlate with clinical measures.
2014	Liu	HAM/TSP *n* = 18 Asymptomatic HTLV1 *n* = 4 MS *n* = 18 Controls *n* = 10	Mean ± SD 11 ± 7	Cross-sectional; case control	N/A	3.0 T	Spinal cord area	Cervical; thoracic; lumbar	No	No	No	N/A	Positive	In HAM/TSP, there was cervical, thoracic and lumbar cord atrophy. There was a similar atrophy pattern in an asymptomatic HTLV-1. In HAM/TSP, the cervical and thoracic atrophy correlated with “Ambulation Index”; and cervical cord atrophy correlated with disease duration.
2017	Taniguchi	HAM/TSP *n* = 15 Controls *n* = 20	Mean ± SD 4.1 ± 3.0 years	Cross-sectional; case control	N/A	3.0 T	Spinal cord area	Cervical; thoracic	No	No	Yes	N/A	Positive	In HAM/TSP, there was reduced cervical and thoracic cord CSA compared with controls. Pathologically, it was more prominent in the WM, especially lateral columns. It did not correlate with clinical metrics.
2014	Vilchez	HAM/TSP *n* = 10 Asymptomatic HTLV1 *n* = 11 Controls *n* = 18	11.9 ± 8.4 years	Cross-sectional; case control	N/A	3.0 T	Spinal cord area Volumetry DTI T2 hyperintensities	Cervical; thoracic	No	No	No	N/A	Positive	In HAM/TSP, there was reduced spinal cord area and volume–most marked in the thoracic cord; focal T2 hyperintensities in the anterolateral and dorsal columns; and reduced FA in the ventral and dorsal tracts compared to asymptomatic HTLV1 and controls. Reduced cervical cord CSA and volume correlated with disease duration; and reduced FA in the dorsal tracts correlated with ASIA score. No cervical cord atrophy or altered DTI parameters were detected in asymptomatic HTLV1.
**Infarct**														
2013	Theaudin	Spinal cord infarct *n* = 2	2 days and 3 days	Longitudinal; case series	Day 3–4; Day 9–10; Day 15–22	1.5 T	DTI	Cervical; thoracic; lumbar	No	No	No	N/A	Positive	In both cases there was initial reduced FA in the spinal cord, that progressively reduced in the case with worsening symptoms and progressively increased in the case with improving symptoms.
**Sensory Neuronopathy**														
2012	Bao	Sensory neuronopathy *n* = 9 (follow-up *n* = 3) Disease controls *n* = 16 (ALS *n* = 14, SACD *n* = 2) Controls *n* = 20	Mean (range): 4.2 (0.5–15) years	Cross-sectional; longitudinal; case control	3 cases at 4, 8, and 14 months	3.0 T	Spinal cord area MEDIC TRIM DRG, posterior column, C7 nerve diameter and signal intensity.	Cervical	No	No	No	N/A	Positive	In sensory neuronopathy, MEDIC and TRIM sequences reveal increased signal intensity and decreased area of DRG and posterior columns, and decreased C7 nerve root area compared to both healthy and disease-control groups. There was no correlation between sensory ataxia and imaging findings.
2016	Casseb	Sensory neuronopathy *n* = 28 (idiopathic *n* = 18; Sjogren’s *n* = 4; Other *n* = 6 (HTLV, autoimmune hepatitis, paraneoplastic, MGUS, VB12 deficiency) Disease controls *n* = 14 (diabetic neuropathy *n* = 14) Controls *n* = 20	11.4 ± 9.3 years	Cross-sectional; case control	N/A	3.0 T	DTIT2 hyperintensities	Cervical	No	No	No	N/A	Positive	In sensory neuronopathy, reduced FA in the cervical cord may differentiate from polyneuropathies or controls. DTI abnormalities preceded T2 hyperintensities and did not correlate with clinical measures.

**Table 6:** Research priorities for viable clinical quantitative spinal applications.

Disease specific spinal consortia Accessible data repositories Age-, sex-, height-stratified normative data from healthy controls Inclusion of several conditions and phenotypes so that spinal signatures of a specific condition is contrasted to disease controls not to healthy controls Data governance frameworks for accessing anonymized radiological data Open-source software and analysis suites Spinal imaging in conditions associated with primary brain pathology (AD, PD, HD, MSA) Integrative combined brain-cord studies No slice gaps Robust machine-learning (ML) initiatives for individual spinal data classification into relevant diagnostic, phenotypic, and prognostic categories Multi-class, multi-label classification instead of binary classification into “disease” versus “healthy” categories Comparison of multiple ML models in a single study and same training data to directly compare model performance and accuracy Validation of ML models with blinded external (off-site) data Multi-cohort studies Assessment of young cohorts for developmental factors Pre-symptomatic longitudinal studies with an emphasis on phenoconversion prediction Genetic data repositories Implementation and “real-life” testing of proposed fMRI, QBI, HARDI, MRS spinal pipelines Comprehensive clinical characterization with the inclusion of relevant covariates, occupation history, environmental factors, and anthropomorphic measures height, Using relevant clinical metrics for radiological correlations (PLSFRS, correct staging framework) Post-mortem correlates of spinal alterations as validation Uniformity in symptoms duration at study entry in longitudinal studies Correlations with wet biomarkers such as relevant CSF markers (neurofilaments, etc.) Correlations to relevant neurophysiology indices (TMS to CST DTI and MUNE MUNIX to GM measures etc.)

## Data Availability

No patient data have been utilized for the drafting of this review paper.

## Supplementary Materials

The following supporting information can be downloaded at: https://www.mdpi.com/article/10.3390/biology13110909/s1, Supplementary File S1: PRISMA Checklist.

## Abbreviations

*ABCD1*—ATP binding cassette subfamily D member 1; ACRA—autosomal recessive cerebellar ataxia; AD—autosomal dominant; ADNI—Alzheimer’s Disease Neuroimaging Initiative; ALD—adrenoleukodystrophy; ALS—amyotrophic lateral sclerosis; ALSFRS—amyotrophic lateral sclerosis functional rating scale; ALSFRS-R—revised amyotrophic lateral sclerosis functional rating scale; ASIA—American Spinal Cord Injury Association; ATXN2—ataxin-2; AUC—area under the receiving operating characteristic curve; AxD—axial diffusivity; C9orf72—chromosome 9 open reading frame 72; CALSNIC—Canadian ALS Neuroimaging Consortium; Cho/Cr—choline to creatine ratio; cHSP—complicated HSP; CSA—cross-sectional area; CST—corticospinal tracts; DRG—dorsal root ganglion; DTI—diffusion tensor imaging; FA—fractional anisotropy; FARS—Friedreich’s ataxia rating scale; FDRA—Friedreich’s ataxia; fMRI—functional MRI (fMRI); FoV—field of view; FTD—frontotemporal dementia; FUS—fused-in sarcoma; FVC—forced vital capacity; GENFI—The Genetic Frontotemporal Initiative; GM—grey matter; GRASE—gradient and spin echo sequence; HARDI—high angular resolution diffusion imaging; HAM/TSP—HTLV1 associated myelitis/tropical spastic paraparesis; HD—Huntington’s disease; HSP—hereditary spastic paraparesis; HTLV1—human T-lymphotropic virus 1; ihMTR—inhomogeneous magnetization transfer ratio; IQR—interquartile range; LMND—lower motor neuron disease; ML—machine learning; MEDIC—multiple echo data image combination; MD—mean diffusivity; MGUS—monoclonal gammopathy of uncertain significance; mIns/Cr—myo-inositol to creatine ratio; MMT—manual muscle test; MRI—magnetic resonance imaging; MND—motor neuron disease; MRS—magnetic resonance spectroscopy; MS—multiple sclerosis; MTR—magnetization transfer ratio; MWI—myelin water imaging; NAA/Cho-N—acetyl aspartate to choline ratio; NAA/Cr-N—acetyl aspartate to creatine ratio; NAA/mIns-N—acetyl aspartate to myo-inositol ratio; NiSALS—Neuroimaging Society in ALS; NMO—neuromyelitis optica; NODDI—neurite orientation dispersion and density imaging; OPTN—optineurin; pHSP—pure HSP; PLS—primary lateral sclerosis; PMA—progressive muscular atrophy; PPMS—primary progressive multiple sclerosis; PPS—post-polio syndrome; PRISMA—preferred reporting items for systematic reviews and meta-analyses; PSIR—phase sensitive inversion recovery; QBI-q—ball imaging; qMT—quantitative magnetization transfer; QSM—quantitative susceptibility mapping; RD–radial diffusivity; ROI–region of interest; RRMS—relapsing remitting multiple sclerosis; SACD—subacute combined degeneration; SARA—scale for the assessment and rating of ataxia; SBMA–spinal bulbar muscular atrophy; SCA—spinocerebellar ataxia; SD—standard deviation; SMA—spinal muscular atrophy; SMN1—survival of motor neuron 1; SOD1–superoxide dismutase type 1; SPG—spastic paraplegia; SPRS—spastic paraplegia rating scale; SYNE1—synaptic nuclear envelope protein 1; T—Tesla; TARDBP—TAR DNA binding protein; TBK1—TANK binding kinase 1; tNAA/m-Ins—total N-acetyl aspartate-to-myo-inositol ratio; TRIM—turbo inversion recovery magnitude; VAPB—vesicle-associated membrane protein-associated protein B/C; VB12—vitamin B12; WM—white matter.
